# Increased Efficiency of Current‐Induced Motion of Chiral Domain Walls by Interface Engineering

**DOI:** 10.1002/adma.202007991

**Published:** 2021-02-04

**Authors:** Yicheng Guan, Xilin Zhou, Tianping Ma, Robin Bläsing, Hakan Deniz, See‐Hun Yang, Stuart S. P. Parkin

**Affiliations:** ^1^ Max Planck Institute for Microstructure Physics Weinberg 2 Halle (Saale) D‐06120 Germany

**Keywords:** dusting layers, Dzyaloshinskii–Moriya interaction, racetrack memory, synthetic antiferromagnets

## Abstract

Magnetic racetrack devices are promising candidates for next‐generation memories. These spintronic shift‐register devices are formed from perpendicularly magnetized ferromagnet/heavy metal thin‐film systems. Data are encoded in domain wall magnetic bits that have a chiral Néel structure that is stabilized by an interfacial Dzyaloshinskii–Moriya interaction. The bits are manipulated by spin currents generated from electrical currents that are passed through the heavy metal layers. Increased efficiency of the current‐induced domain wall motion is a prerequisite for commercially viable racetrack devices. Here, significantly increased efficiency with substantially lower threshold current densities and enhanced domain wall velocities is demonstrated by the introduction of atomically thin 4d and 5d metal “dusting” layers at the interface between the lower magnetic layer of the racetrack (here cobalt) and platinum. The greatest efficiency is found for dusting layers of palladium and rhodium, just one monolayer thick, for which the domain wall's velocity is increased by up to a factor of 3.5. Remarkably, when the heavy metal layer is formed from the dusting layer material alone, the efficiency is rather reduced by an order of magnitude. The results point to the critical role of interface engineering for the development of efficient racetrack memory devices.

## Graphical abstract

The efficiency of current‐induced domain wall motion in both ferromagnetic/heavy metal and synthetic antiferromagnet systems is significantly enhanced via the insertion of atomically thin dusting layers at the ferromagnet/heavy metal interface. The enhancement is due to the tuning of the strength of the Dzyaloshiskii–Moriya interaction and the effective magnetic uniaxial anisotropy energy.

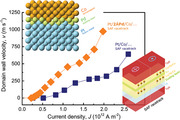

## 1 Introduction

Solid‐state spintronic devices are recognized as one of the most promising candidates to enable “beyond CMOS” technologies for solid‐state data storage, logic, and neuromorphic computing applications.^[^ ^1^, ^2^ ^]^ The operation of these devices is primarily based on the generation and manipulation of spin‐polarized currents. These currents can be used, in particular, to manipulate magnetic bits that are encoded within magnetic spin textures (domains,^[^ ^3^ ^]^ skyrmions,^[^ ^4^ ^]^ or antiskyrmions^[^ ^5^ ^]^) in nanoscale racetracks. A racetrack‐based memory is fundamentally a shift register in which the fast and energy‐efficient motion of such magnetic bits along 2D or 3D racetracks by spin current is crucial for its commercial implementation.^[^ ^3^, ^6^, ^7^ ^]^

Current‐induced domain wall (DW) motion (CIDWM) has significantly evolved from in‐plane magnetic^[^ ^8^ ^]^ to synthetic antiferromagnetic (SAF)^[^ ^9^, ^10^ ^]^ racetracks with advances in volume spin‐transfer torque (STT)^[^ ^11^, ^12^ ^]^ and spin‐orbit torque (SOT)^[^ ^13^, ^14^, ^15^, ^16^ ^]^ mechanisms. Driven by a chiral spin torque that arises from the spin‐orbit coupling in the presence of broken inversion symmetry at ferromagnet/heavy metal (HM) interfaces,^[^ ^17^ ^]^ Néel DWs in thin films with strong perpendicular magnetic anisotropy (PMA), stabilized by a Dzyaloshinskii–Moriya interaction (DMI) at the ferromagnet/HM interfaces,^[^ ^18^ ^]^ can be moved along the current direction at high velocities,^[^ ^12^, ^15^, ^19^ ^]^ in both straight and curved racetracks.^[^ ^20^ ^]^ An even more efficient DW motion was reported in SAF racetracks that are composed of two perpendicularly magnetized ferromagnetic sub‐racetracks coupled antiferromagnetically across an ultrathin ruthenium layer.^[^ ^10^ ^]^ The giant exchange coupling torque (ECT) in the SAF structure provides an additional dominating driving mechanism that allows for an increased DW propagation velocity beyond ≈1000 m s^−1^.^[^ ^10^, ^21^ ^]^ The ECT in rare earth‐transition metal alloys is further maximized at the angular momentum compensation temperature of the ferrimagnetic alloy.^[^ ^22^, ^23^ ^]^ Recently, efficient CIDWM was also found in certain magnetic insulators.^[^ ^24^ ^]^

Significant progress has been made regarding a detailed understanding of the interface derived chiral spin torque^[^ ^19^ ^]^ and magnetic chirality^[^ ^25^ ^]^ with respect to the underlying mechanisms of CIDWM,^[^ ^26^ ^]^ for example, by varying the composition of the HM underlayer and capping layer that is in contact with the interface ferromagnetic layer^[^ ^19^, ^27^ ^]^ or by tuning the thickness of the ferromagnetic layers.^[^ ^15^, ^28^ ^]^ The notion of interfacial dusting layers (DL) was initially proposed to demonstrate the interfacial origin of the giant magnetoresistance (GMR) effect, in which atomically thin ferromagnetic layers were inserted at the interfaces in sandwich structures.^[^ ^29^ ^]^ There has been significant interest in the insertion of DLs in several studies primarily related to the switching of the magnetic moment of micro‐elements.^[^ ^30^ ^]^ Here, we show that by introducing atomically thin dusting layers of selected 4d and 5d HMs at the ferromagnet/platinum interface, a significant enhancement of the efficiency of the CIDWM is achieved for both single magnetic layer and SAF racetrack structures. The Néel DWs move up to more than three times faster, for the same injected current density, compared to otherwise identical structures without any DL. Moreover, the threshold current density, *J*_th_, defined as the minimum current density required to overcome effective pinning fields and move the DWs, can be substantially reduced by incorporating atomically thin DLs.

## 2 Results and Discussion

**Figure 1:**
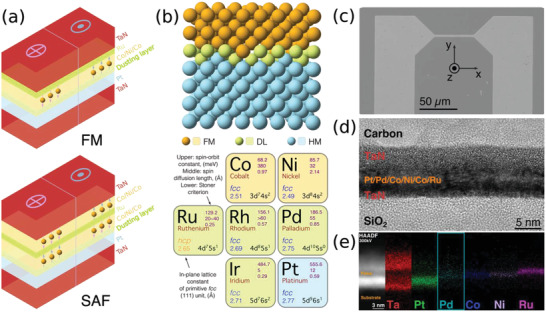
Engineered FM and SAF racetrack structures with interfacial dusting layers. a) Schematic representation of the FM (top) and SAF (bottom) racetrack structures with a dusting layer (DL) inserted between the heavy metal (Pt) and ferromagnetic metal (Co) layers. b) Top: Schematic illustration of the atomic stack of the FM/DL/HM structure along the fcc (111) direction. Bottom: The color‐coded elements employed in the FM/DL/HM stack; orange, green, and light blue correspond to FM, DL, and HM, respectively. The numbers in the upper right position of each element square correspond to (from top to bottom): the spin‐orbit coupling constant, spin diffusion length, and Stoner criterion parameter, respectively. The number in the lower‐left position is the in‐plane lattice constant of the corresponding fcc (111) unit. c) Scanning electron microscopy of a typical racetrack. d) Cross‐sectional HRTEM image, and e) the corresponding EDX mapping of an FM film with 1 Å thick Pd dusting layer, in which the presence of the atomically thin Pd layer is highlighted.

Two sets of structures were prepared by DC magnetron sputtering at room temperature, as shown in **Figure** **1**a a ferromagnetic structure consisting of Co (3 Å)/Ni (7 Å)/Co (1.5 Å) sandwiched between a Pt (15 Å) underlayer and an Ru (8.5 Å) capping layer, hereafter referred to as an FM structure; and a synthetic antiferromagnet structure deposited on the same Pt (15 Å) underlayer and consisting of a lower ferromagnetic layer of Co (3 Å)/Ni (7 Å)/Co (1.5 Å) and an upper ferromagnetic layer of Co (5 Å)/Ni (7 Å)/Co (1.5 Å) antiferromagnetically exchange‐coupled through an Ru (8.5 Å) spacer, hereafter referred to as an SAF structure. A series of atomically thin layers (hereafter referred to as dusting layers, DL) of Pd, Ir, Rh, and Ru with thicknesses varying from 1 to 7 Å are inserted directly onto the Pt underlayer in both structures before the ferromagnetic materials are deposited. Schematic images of the FM and SAF structures are shown in Figure 1a with the elemental DLs illustrated in Figure 1b. The CIDWM was studied in racetracks of 3 µm wide and 50 µm long, which were fabricated by photolithography and Ar ion milling (Figure 1c).^[^ ^10^, ^15^, ^19^, ^22^, ^31^, ^32^ ^]^ The motion of individual DWs in these nanowires in response to voltage pulses of a fixed length (≈10 ns) was detected using Kerr microscopy.^[^ ^10^, ^15^, ^19^ ^]^ The DW positions in the nanowire before and after the pulse injection are recorded and, thereby, used to determine the DW velocity along the racetrack.

A cross‐sectional high‐resolution transmission electron microscopy (HRTEM) image of an FM structure with an ≈1 Å thick Pd DL is shown in Figure 1d. The image presents a highly (111) oriented structure of the face‐centered‐cubic (fcc) thin‐film structure. The smooth surface of the films is confirmed by atomic force microscopy imaging (see Figure S1, Supporting Information). The high‐angle annular dark‐field scanning TEM (HAADF‐STEM) image and the associated energy‐dispersive X‐ray spectrometry (EDX) maps of the layered structure in Figure 1e show that the Pd DL is at the expected location between the Pt layer and the Co/Ni/Co layer, although the EDX signal is obviously very weak, and moreover, is broadened due to scattering processes and roughness of the individual layers.^[^ ^33^ ^]^

**Figure 2:**
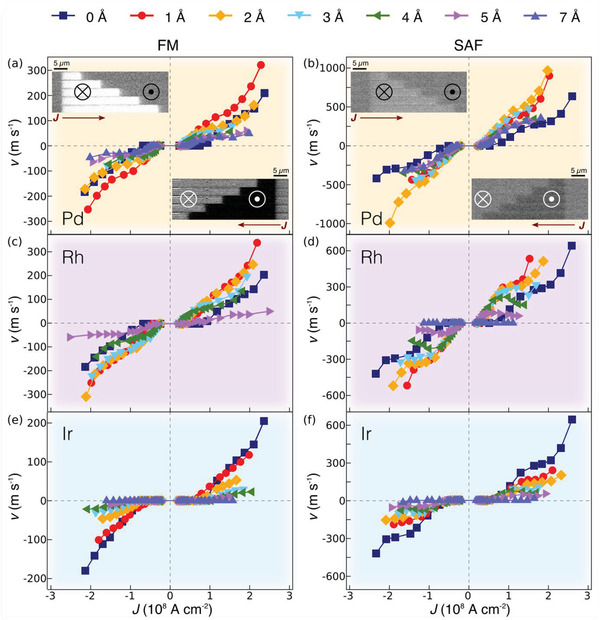
Interfacial DL engineered chiral domain wall motion in FM and SAF structures. a–f) Current‐induced DW motion in the FM (left) and SAF (right) structures with various DL materials: Pd ((a) and (b), orange background), Rh ((c) and (d), violet background), and Ir ((e) and (f), blue background). The insets in (a) and (b) illustrate typical Kerr images of the domain wall motion in response to a series of injected current pulses (≈1.0 × 10^8^ A cm^−2^) composed of, respectively, twelve (a) and four (b) 10 ns long pulses in the FM and SAF samples with 1 Å Pd DL, that confirms that all the DWs move in the direction of current injection. Differences in image contrast originate from the magnetization difference in the FM and SAF samples. The bright and dark parts correspond to down (⊗ or ↓) and up (⊙ or ↑) domains. The thickness of the inserted dusting layers is varied from 0 Å (navy squares), 1 Å (red circles), 2 Å (orange diamonds), 3 Å (light blue triangles), 4 Å (olive triangles), 5 Å (purple triangles), and 7 Å (orchid triangles).

Distinct CIDWM behaviors are observed for the FM and SAF structures that depend sensitively on the DL material and thickness. Representative examples of CIDWM for DLs composed of Pd, Rh, and Ir are shown in **Figure** **2**. In all these cases the DWs move along the direction of injected current, independent of the DL thickness. For the case of a sufficiently thick Ru DL, however, the DWs are driven along the electron flow direction, as discussed in the Supporting Information (see Section S10 and Figure S16, Supporting Information). For the case of both Pd and Rh DLs, *J*_th_ is significantly decreased in the FM structure for DLs as thin as only 1 Å (Figure 2a,c), and the DW velocity, *v*, is increased for all current densities considered. For thicker DLs *v* is lower. The maximum current density that can be applied to the racetracks is limited by the formation of multiple magnetic domains that we attribute to an increase in temperature of the nanowire, as has previously been observed in nanowires of comparable resistance.^[^ ^31^ ^]^ By contrast, for the SAF structure, the performance of the CIDWM is improved for all Pd DL thicknesses considered and is maximized for a Pd DL that is just ≈2 Å thick. The DW velocity is increased to ≈1000 m s^−1^ (Figure 2b), which is ≈3.5 times higher than that of the reference SAF at the same current density. For the Rh DL case the DW velocity is also increased for ultra‐thin DLs (1 and 2 Å) but the maximum *v* achieved was lower (≈600 m s^−1^).

A significantly different behavior is observed when an Ir DL is employed. In the FM case *J*_th_ drops when the DL is 1 Å thick, and then increases almost linearly with further increases in Ir thickness (Figure 2e). Only a small enhancement in *v* was observed at low current densities for 1 Å Ir, but otherwise the CIDWM performance was worse than the reference FM sample. For the SAF structure, a systematic deterioration of the CIDWM occurs as soon as an Ir DL is inserted (Figure 2f). However, it is worth noting that *J*_th_ drops smoothly for Ir DLs with thicknesses up to 5 Å but then increases dramatically for thicker layers.

**Figure 3:**
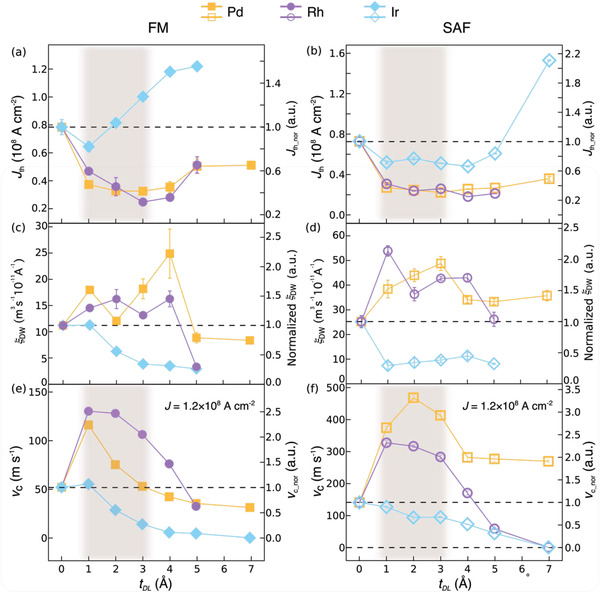
DL thickness dependence of the DW velocity (*v*) and threshold current density (*J*_th_). a,b) The threshold current density (*J*_th_) as a function of DL thickness in the FM and SAF samples. The dashed lines represent the threshold current density of the reference samples. The right axis represents the normalized threshold current density (*J*_th_nor_) with respect to the reference samples. c,d) The DW mobility (ξ_DW_) around the threshold current in the FM and SAF samples for various DL thicknesses (*t*_DL_). The dashed lines represent the DW mobility of the reference samples. e,f) The DW velocity *v*_C_ at a fixed current density (≈1.2 × 10^8^ A cm^−2^) in the FM and SAF samples with various DL thicknesses (*t*_DL_). The dashed lines represent the velocity of the reference samples. The right axis is the normalized DW velocity (*v*_c_nor_) with respect to the reference samples. The colored regions illustrate the interfacial DL thickness range, where the efficiency of CIDWM is maximized. Pd, Rh, and Ir DL correspond to orange squares, violet circles, and light blue diamonds, respectively. The filled and open symbols correspond to the FM and SAF cases, respectively.

To directly compare the influence of different DLs on the CIDWM performance, i) *J*_th_, ii) the DW mobility ξ_DW_ near *J*_th_, which is derived from the slope of *v* versus current density *J*, and, iii) *v*_C_ = *v* at *J* = 1.2 × 10^8^ A cm^−2^ are plotted as a function of DL thickness *t*_DL_ in **Figure** **3**a–f for both the FM and SAF structures. To distinguish CIDWM from DW creep that occurs even at tiny current densities,^[^ ^34^ ^]^ we define *J*_th_ as the current density above which *v* exceeds 5 m s^−1^. For both the FM and SAF cases, for Pd and Rh DLs, a decreased *J*_th_ together with an increased ξ_DW_ is observed for thin DLs (1–4 Å), as shown in Figure 3a–d. A decrease in *J*_th_ of up to 30% and an increase in ξ_DW_ of more than 200% is found for both the FM and SAF cases. For Ir DLs, although a slight decrease in *J*_th_ is found for the thinnest DLs, substantial decreases in ξ_DW_ are observed for both FM and SAF structures. For Pd and Rh DLs the dependence of *v*_C_ on *t*_DL_ is similar for both the FM and SAF cases: *v*_C_ initially increases significantly and then drops monotonically as *t*_DL_ is increased from zero; by comparison, a monotonic drop in DW velocity is observed with increasing *t*_DL_ for Ir DLs (Figure 3e,f).

**Figure 4:**
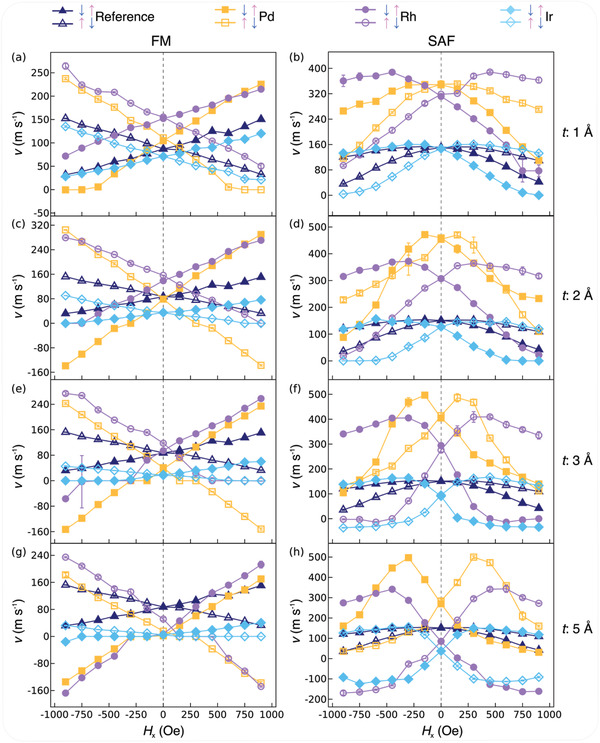
Longitudinal magnetic field dependence of DW velocity on the dusting layer. a–f) The longitudinal field dependence of DW velocity (*v*–*H* *_x_*) at a fixed current density (≈1.2 × 10^8^ A cm^−2^) in the FM (left) and SAF (right) structures. The open and filled symbols represent the ↑↓ and ↓↑ domain configurations, respectively. The samples with DL thicknesses of 1 Å (a,b), 2 Å (c,d), 3 Å (e,f), and 5 A (g,h) are shown. Other DL thicknesses are included in the Supporting Information (Figures S2 and S3, Supporting Information). The reference samples and those with Pd, Rh, and Ir DL are represented by navy triangles, orange squares, violet circles, and light blue diamonds, respectively.

**Figure 5:**
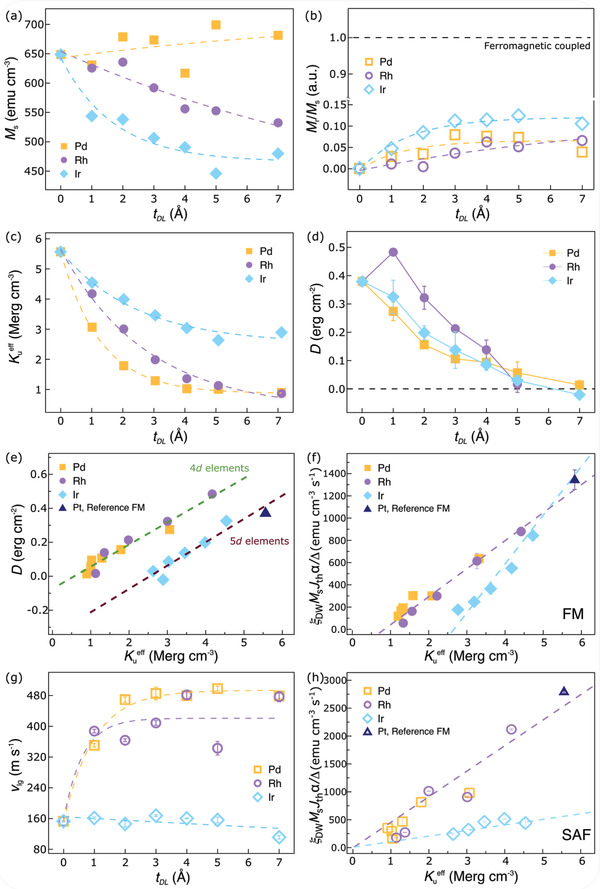
Interfacial dusting layer engineered magnetic properties. a–c) The DL thickness dependence of: (a) saturation magnetization of the FM structures, (b) remnant magnetization to saturation magnetization ratio for the SAF structures, (c) effective uniaxial anisotropy constant $K_{u}^{\text{eff}}$ for the FM structures, d) *D* calculated from the FM cases. e) *D* of FM samples plotted as a function of $K_{u}^{\text{eff}}$, in which the dashed lines are linear fits for the groups of DLs formed from 4d elements (Pd and Rh, olive) and 5d elements (Ir and Pt, brown), respectively. The Pt data point corresponds to the reference sample without any DL. f,h) Dependence of *αξ*_DW_ *M_S_J*_th_/Δ on $K_{u}^{\text{eff}}$ for FM and SAF cases, respectively. The dashed lines are linear fits for the groups of DLs formed from 4d elements (Pd and Rh, violet) and 5d elements (Ir and Pt, light blue), respectively. The Pt data point corresponds to the reference sample without any DL. g) DL thickness dependence of the DW peak velocity *v*_lg_ extracted from the *v–H*_*x*_ curves for the SAF structures in Figure 4 and Figure S3, Supporting Information. The filled and open symbols correspond to the FM and SAF cases, respectively.

The CIDWM is derived from a chiral spin torque, in which the chirality of the DWs in both the FM and SAF structures is stabilized by an interfacial DMI arising from HM underlayers with strong spin‐orbit coupling.^[^ ^15^ ^]^ Thus *v* depends sensitively on magnetic fields applied along the racetrack:^[^ ^10^, ^15^, ^19^ ^]^ external longitudinal magnetic fields *H* *_x_* add or subtract from the DMI effective fields that stabilize the chiral DWs. *v* was measured as a function of *H* *_x_*. The movement of DWs with both ↓↑ and ↑↓ domain configurations with respect to *H* *_x_* under positive current are shown in **Figure** **4** in which racetracks incorporating Pd, Rh, and Ir DLs with thicknesses of 1, 2, 3, and 5 Å are presented. Data are shown at a fixed current density (*J* = 1.2 × 10^8^ A cm^−2^). Results for other DL thicknesses are given in the Supporting Information (Figures S2 and S3, Supporting Information). For the FM structure *v* depends linearly on *H* *_x_* ^[^ ^15^, ^19^, ^22^ ^]^ with a slope that strongly depends on the DL material and thickness, and whose sign reverses for ↓↑ and ↑↓ DWs. This behavior can be readily understood within a 1D DW analytical model of the chiral SOT:^[^ ^15^, ^19^, ^35^ ^]^ there are two key characteristics: the slope of the *v*–*H* *_x_* curve, and the magnitude of *H* *_x_* where *v* = 0. By fitting these curves, the interfacial DMI strength *D* ^[^ ^19^, ^35^, ^36^ ^]^ can be determined: the values of *D* thereby derived are given in **Figure** **5**d. For the SAF case, the *v*–*H* *_x_* curve has a more complex profile^[^ ^10^, ^21^, ^32^ ^]^ but which can be understood within a 1D model that includes the ECT in addition to the SOT. The detailed fits to the curves given in Figure 4b,d,f,h are discussed in the Supporting Information (Figures S4–S9, Supporting Information). Although the 1D model makes some assumptions, in particular, that the form and width of the DW does not change during its motion, and that the predominant SOT is damping‐like, nevertheless such a model has proven to be highly successful in accounting for the CIDWM in many previous studies and in unravelling the underlying physics.^[^ ^10^, ^15^, ^19^, ^21^, ^23^, ^24^, ^35^, ^36^ ^]^

In order to interpret the influence of the DLs on the CIDWM, i) the saturation magnetization *M*_s_, ii) the effective perpendicular magnetic anisotropy constant $K_{u}^{\text{eff}}$ (defined as 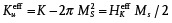, where *K* is the perpendicular magnetic anisotropy, and $H_{K}^{\text{eff}}\;$ is the hard axis anisotropy field), and iii) the ratio of the remnant magnetization (magnetization at ≈0 T, *M*_r_) to saturation magnetization (magnetization field ≈ 1.5 T) *M*_r_/*M*_s_ in the SAF structures, were measured. The dependence of these parameters on *t*_DL_ are plotted in **Figure** **5**a,c. A monotonic drop in $K_{u}^{\text{eff}}$ can be observed with increasing *t*_DL_ in all cases (Figure 5c). *M*_s_ varies little with Pd DL, while a monotonic drop for the Ir and Rh DLs cases is observed (Figure 5a). Proximity induced magnetic moments (PIM) in heavy metals can contribute considerably to the *M*_s_ of ferromagnet/HM systems.^[^ ^19^, ^37^ ^]^ Based on the Stoner criterion (Figure 1b), it would not be surprising if the PIM decreases when the Ir and Rh DLs are inserted between the Co and Pt layers. On the other hand, we suppose that there may be a considerable PIM in the Pd DL itself since Pd is very close to the Stoner criteria for magnetism.^[^ ^38^ ^]^

To demonstrate that the effect of the DL on the CIDMW is interfacial and is not a volume effect, a set of FM structures was prepared in which a 2 Å DL is systematically moved parallel to the film normal away from the Co/Pt interface into the interior of the Pt underlayer. The influence of the ultrathin DL on the magnetic properties and the CIDWM is clearly effective only when located at or very near (<3 Å) the interface (see Section S11 and Figure S17, Supporting Information).

The dependence of *M*_r_/*M*_s_ on *t*_DL_ is shown in Figure 5b (the associated *M*–*H* loops are plotted in Figure S14 in Section S8, Supporting Information). The changes in *M*_r_/*M*_s_ with *t*_DL_ are predominantly due to the variation of *M*_s_ in the lower sub‐layer of the SAF structure, as found for the FM case in Figure 5a. It has previously been shown that CIDWM in SAF structures is largely derived from a giant ECT. The more similar are the two sub‐layer moments, that is, *M*_r_/*M*_s_ = 0, the larger is the ECT.^[^ ^10^, ^21^, ^32^ ^]^ Note that the reference SAF sample has been optimized so that *M*_r_/*M*_s_ is close to zero. As discussed above, the efficiency of CIDWM is increased with several‐angstrom‐thick DLs even though the introduction of the DL causes *M*_r_/*M*_s_ to deviate from zero (due to changes in the PIM). Thus, even faster CIDWM in the DL engineered racetracks is anticipated when *M*_r_/*M*_s_ is reduced to zero by small modifications to the moments of the upper or lower ferromagnetic layers in the SAF structure.

In the FM structure, a monotonic drop in *D* is observed with increasing *t*_DL_ except for a slight increase for the ≈1 Å thick Rh DL case (Figure 5d). It is worth noting that, as the Ir DL thickness is increased, there is a clear sign change of *D*. For each DL we find that *D* varies linearly with $K_{u}^{\text{eff}}$, as shown in Figure 5e. Interestingly, we find a similar slope for all DLs, but split into two groups with different intercepts corresponding to the 4d elements, on the one hand, and the 5d elements, on the other. Since both parameters depend on the strength of interfacial spin‐orbit coupling, such two distinct groupings of 4d and 5d DLs may be attributed to the different contributions of the orbital angular moments of the 4d and 5d electrons, as revealed both theoretically and experimentally in prior work.^[^ ^39^ ^]^

The *t*_DL_ dependence of the peak DW velocity, *v*_lg_, in the SAF structure with the aid of an external longitudinal field (shown in Figure 4) is summarized in Figure 5g. For the Pd and Rh DLs, *v*_lg_ increases rapidly and then saturates as the DL is thickened. For the Ir DLs, however, *v*_lg_ remains almost constant with a gradual decrease when the DL thickness is increased.

The origin of *J*_th_ in both SOT‐ and ECT‐driven CIDWM is unclear but we can examine the DW threshold spin current density, $\;J_{\text{th}}^{s}=\;J_{\text{th}}\theta_{\text{SH}}$, where θ_SH_ is the effective spin Hall angle. This quantity is proportional to ξ_DW_ *M_S_J*_th_α/Δ, as derived in Sections S4 and S6, Supporting Information. Here, α is the Gilbert damping parameter and is obtained from optical ferromagnetic resonance (OFMR) measurements (see Section S9, Supporting Information). Plots of ξ_DW_ *M_S_J*_th_α/Δ versus $K_{u}^{\text{eff}}$ for both the FM and SAF cases show two distinct linear relationships for 4d DLs, on the one hand, and 5d DLs, on the other hand (Figure 5f,h). Thus, the threshold spin current is linearly related to $K_{u}^{\text{eff}}$ for both the FM and SAF cases and can be reduced by introduction of suitable DLs.

For the practical application of racetrack memory devices, low *J*_th_, high ξ_DW_, and high *v* are required. For the FM case, the linear dependence of $K_{u}^{\text{eff}}$ on *D* shows that once a smaller *J*_th_ is realized through decreasing $K_{u}^{\text{eff}}$, the saturation DW *v* at high current density is also decreased due to a smaller *D*.^[^ ^35^ ^]^ However, at currents just above the threshold current, the DW velocity is higher than otherwise would be the case. By contrast, for the SAF case, a low *J*_th_ and high *v* can both be achieved by decreasing $K_{u}^{\text{eff}}$ since *v* no longer depends on *D* but rather largely on the exchange coupling constant *J*_ex_ (see Section S4, Supporting Information). More importantly, the decreased thermal stability due to the decrease of $K_{u}^{\text{eff}}$ in the FM case can rather be enhanced by the interlayer exchange coupling in the SAF case, resulting in a lower sensitivity of CIDWM to both temperature and the current pulse length (see Section S5, Supporting Information).

## 3 Conclusion

By using DLs of several 4d and 5d materials, we have demonstrated a significantly enhanced CIDWM performance for both FM and SAF racetracks. Notably, up to an ≈250% increase in DW velocity and an ≈70% decrease in threshold current density was found by the insertion of just one monolayer thick DLs of Pd at the Pt/Co interfaces. Most interestingly, the materials from which the DLs are formed, for example Pd, when used as a replacement for the entire Pt underlayer perform badly and, thereby, highlights the critical role of atomic interfacial engineering in racetracks.^[^ ^19^ ^]^ We have also revealed a systematic dependence of DMI on the effective uniaxial anisotropy $K_{u}^{\text{eff}}$ in systems with DLs. The newly defined DW threshold spin current density and its relationship with $K_{u}^{\text{eff}}$ is very useful for the further improvement of the CIDWM efficiency. We note that these conclusions could only be reached by preparing sets of identical samples with DL variations on the angstrom length scale. Our studies show how important is such atomic interfacial engineering and broaden the selection of materials for applications in racetrack devices.

## 4 Experimental Section

### Sample Preparation and DW Velocity Measurement

The thin film samples used in this work were deposited by magnetron sputtering at room temperature on Si wafers that were covered with a thermally oxidized SiO_2_ layer (≈300 Å). All samples were sandwiched between a bottom TaN layer (≈20 Å) and a capping TaN layer (≈50 Å) that had high resistivities. The deposition parameters were calibrated in situ using quartz crystal microbalances and ex situ using X‐ray reflection. The 3  µm × 50 µm racetrack nanowires were fabricated using photolithography and argon‐ion milling. All the injected current pulses had a fixed duration of ≈10 ns. The DW velocities were determined from Kerr microscopy measurements. The DWs were created in the racetrack nanowires by injecting pulses of current in the presence of external longitudinal magnetic fields. In actual devices, it was noted that generating DWs by alternative external field‐free methods would be favored.^[^ ^40^ ^]^

### TEM Specimen Preparation and Investigation

Cross‐sectional TEM specimens were formed by conventional preparation methods using mechanical polishing (both sides), dimple‐grinding (one side) and thinned down for electron transparency by polishing with Ar ions at 5 kV in a Gatan PIPS (precision ion polishing system) system. For HRTEM/STEM investigations, an FEI TITAN 80–300 electron microscope with a probe corrector was used at an accelerating voltage of 300 kV. The EDX experiments were performed with a Super‐X detector system (4 silicon drift detectors placed symmetrically around the sample area inside the objective lens) installed on the microscope for improved efficiency of X‐ray collection. Acquired EDX maps were analyzed and processed by Bruker Esprit software.

### Magnetic Property Measurements and Calculation of DMI Constant *D*

The sample magnetization was measured in a superconducting quantum interference device (SQUID) magnetometer at room temperature. $H_{K}^{\text{eff}}$, the field where the magnetization is rotated from out‐of‐plane to in‐plane, was measured using a vibrating sample magnetometer. *M*_r_ and *M*_s_ of the SAF samples were determined at fields of 0 Oe and 15 kOe, respectively, from out‐of‐plane *M–H* curves (Figure S14, Supporting Information). The Gilbert damping parameter α was obtained from Optical Ferromagnetic Resonance measurements. The DW width was calculated from $\Delta \;=\sqrt{A/K_{u}^{\text{eff}}}\;$ with *A*, the exchange stiffness, set to be a constant of 1.0 μerg cm^−1^.^[^ ^32^ ^]^ The DMI constant *D* was calculated from the expression *D*  = μ_0_ *M_s_* Δ*H*_DMI_^[^ ^35^ ^]^ where *H*_DMI_ was determined from the dependence of the DW velocity on an in‐plane magnetic field.

## Conflict of Interest

The authors declare no conflict of interest.

## Supporting information

Supporting Information

## Data Availability

The data that support the findings of this study are available from the corresponding author upon reasonable request.
