# Supplementary material for: Increased Efficiency of Current‐Induced Motion of Chiral Domain Walls by Interface Engineering
Source: Adv Mater. 2021 Feb 4;33(10):2007991. doi: 10.1002/adma.202007991 (PMC11468057; doi:10.1002/adma.202007991)
Supplement: Supplementary file 1 — Supporting Information [file ADMA-33-2007991-s001.pdf]

© 2021 Wiley-VCH GmbH

ADVANCED MATERIALS

Supporting Information

for *Adv. Mater.*, DOI: 10.1002/adma.202007991

Increased Efficiency of Current-Induced Motion of Chiral Domain Walls by Interface Engineering

*Yicheng Guan, Xilin Zhou, Tianping Ma, Robin Bläsing, Hakan Deniz, See-Hun Yang, and Stuart S. P. Parkin**

© 2021 Wiley-VCH GmbH

Supplementary Information

Increased efficiency of current induced motion of chiral domain walls by interface engineering

*Yicheng Guan, Xilin Zhou, Tianping Ma, Robin Bläsing, Hakan Deniz, See-Hun Yang, and Stuart S. P. Parkin***

This file includes:

Section 1-11

Figures S1-S17

Table S1

Note that the following abbreviations are used in the main text:

FM: single layer racetrack structure

SAF: synthetic antiferromagnetic racetrack structure

DL: dusting layer

CIDWM: current induced domain wall motion

DW: domain wall

DMI: Dzyaloshinskii-Moriya interaction

HM: Heavy Metal

PMA: perpendicular magnetic anisotropy

M_r: remnant magnetization at zero magnetic field

M_s: saturation magnetization

Section 1: Characterization of sample roughness

In Figure 1d of the main text, a well crystallized FM structure with an atomically thin DL was examined by cross-sectional transmission electron microscopy (TEM). Atomic force microscopy (AFM) was also used to characterize the surface roughness of the films. AFM results for typical FM and SAF structures with and without DLs are given in Figure S1a-S1d. The root mean square roughness (R_q) values (averaged over an area of ~2×2 μm^2) are smaller than 2 Å in each case, establishing that surfaces of the samples are atomically smooth.

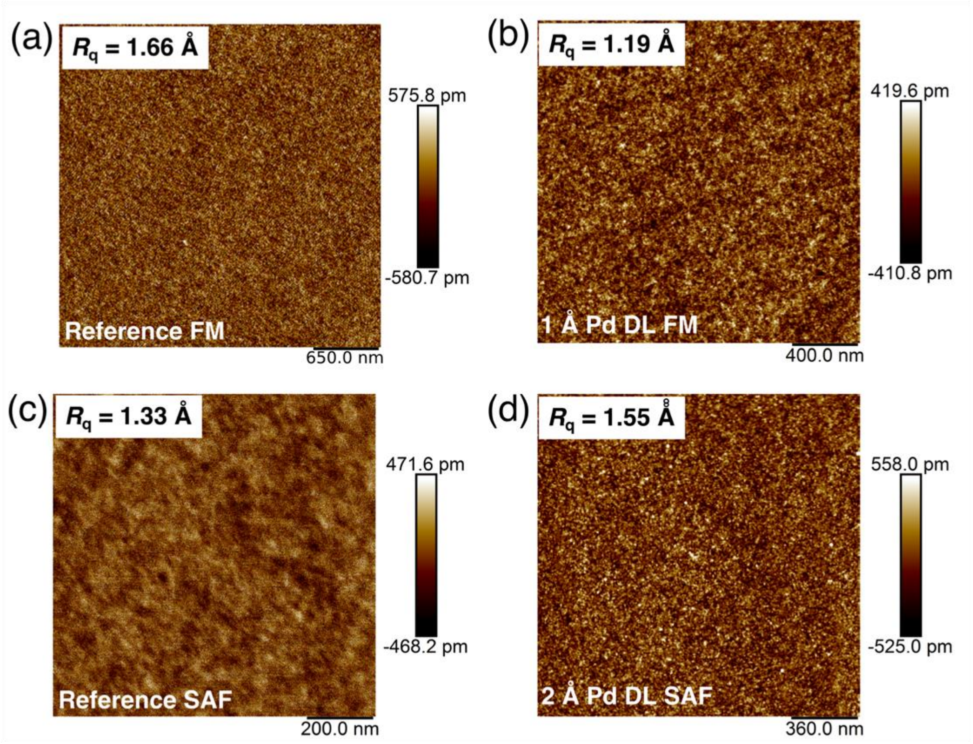

Figure S1 AFM topographic images of typical films with and without interfacial DLs. a) reference FM film without a DL: 20 TaN|15 Pt|3 Co|7 Ni|1.5 Co|8.5 Ru|50 TaN; b) FM film with a 1 Å thick Pd DL: 20 TaN|15 Pt|1 Pd|3 Co|7 Ni|1.5 Co|8.5 Ru|50 TaN; c) Reference SAF film: 20 TaN|15 Pt|3 Co|7 Ni|1.5 Co|8.5 Ru|5 Co|7 Ni|1.5 Co|50 TaN; d) SAF film with a 2 Å thick Pd DL: 20 TaN|15 Pt|2 Pd|3 Co|7 Ni|1.5 Co|8.5 Ru|5 Co|7 Ni|1.5 Co|50 TaN. All thicknesses are given in angstroms. The root mean square roughness R_q of each of the films is given in the image.

Section 2: 1-D analytical model for CIDWM in FM structures and determination of the DMI exchange constant

Figure S2 shows the longitudinal field dependence of the CIDWM for several FM film structures with various DLs. The magnetization profile inside a DW of a 1-D perpendicularly magnetized sample can be expressed as follows: $\theta = 2\tan^{-1}[\exp(\pm\frac{x-q}{\Delta})]$ (Ref. S1). Here, θ is the angle that the magnetization $\boldsymbol{M}$ deviates from the z-direction (out of plane of the racetrack), q is the DW position along the racetrack, $\Delta = \sqrt{A/K_u^{\text{eff}}}$ is the DW width, and $\pm$ correspond to ↓↑ or ↑↓ DW configurations.

The DW magnetization dynamics can be expressed according to the following Landau-Lifschitz-Gilbert (LLG) equation[S4]:

$$\frac{\partial \boldsymbol{M}}{\partial t} = -\gamma \boldsymbol{M} \times \boldsymbol{H}_{\text{eff}} + \frac{\alpha}{m}\boldsymbol{M} \times \frac{\partial \boldsymbol{M}}{\partial t} - b_{\text{J}}\boldsymbol{M} \times \boldsymbol{M} \times \frac{\partial \boldsymbol{M}}{\partial t} - \beta b_{\text{J}}\boldsymbol{M} \times \frac{\partial \boldsymbol{M}}{\partial x} + H_{\text{SHE}}\boldsymbol{M} \times \boldsymbol{M} \times \hat{y}$$

where $\boldsymbol{M}(\boldsymbol{r}, t)$ is the local magnetization vector. The first two terms are the field-like and damping-like torques in the presence of an external field, $\boldsymbol{H}_{\text{eff}}$, the third and fourth terms are the Spin-Transfer Torque (STT) from the polarized current injection, and the last term is the Spin-Orbit Torque (SOT) exerted via spin currents generated from the spin Hall effect in the Pt underlayer as the current is injected along the wire length. Here γ is the gyromagnetic ratio; α is the damping parameter of the ferromagnetic layer; β is the non-adiabatic parameter for spin transfer torque and $b_{\text{J}} = J\frac{\mu_B P}{eM_s}$, where μ_B is the Bohr magneton, e is the electron's charge and P is the polarization rate from the FM layer.

Thus, we derive the following equations:

$$\begin{cases} \alpha\dot{q} \pm \Delta\dot{\psi} = -\beta u \mp \frac{\pi}{2}\gamma\Delta H_{\text{SHE}}\cos(\psi) - \frac{\gamma\Delta}{M_s}V_{\text{pinning}} \\ \dot{q} \mp \alpha\Delta\dot{\psi} = \mp\frac{\gamma\Delta H_k}{2}\sin(2\psi) - u \pm \frac{\pi}{2}\gamma(H_x - H_{DMI})\sin(\psi) \end{cases}$$

where q is the position of the DW, ψ is the angle of magnetization of DW to the longitudinal direction, $u = \frac{\mu_B P J_m}{eM_s}$ is the STT related DW velocity, μ_B is the Bohr magneton, M_s is the saturation magnetization, e is the electron charge, P is the spin polarization of the current and J_m is the current density flowing in the magnetic layer. The spin Hall effective field can be written as $H_{\text{SHE}} = \frac{\hbar\theta_{\text{SH}}J_{Pt}}{2eM_s t}$, where θ_{SH} is the effective spin Hall angle from the DL/Pt bilayer,

t is the thickness of the magnetic layer and $J_{\mathrm{Pt/DL}}$ is the current density flowing in the DL/ Pt bilayer. The DMI effective field can be written as, $H_{\mathrm{DMI}} = \frac{D}{u_0 M_s \Delta}$, where D is the DMI constant. H_k is the shape anisotropy field of the DW and is described by the expression: $H_K = -4\pi t M_S/(t+\Delta)$. The $+$ and $-$ symbols corresponds to the ↑↓ and ↓↑ domain wall configurations.

By setting $\dot{\psi} = 0$ we can calculate the DW velocity for a given current density and external magnetic field. Note that, naively, $\dot{q}$, the DW velocity, will increase with larger Δ and smaller α. Neglecting the STT term, the DW will have a Néel wall configuration so that the SOT is non-zero (the SOT term goes to zero for a Bloch like DW). Thus, the DW velocity will drop to zero when an external field, H_x, that is applied longitudinally along the wire, exactly balances the DMI effective field. We plot v-H_x curves for each sample in Figure S2. By linearly fitting these curves near the crossing point where the DW velocity goes to zero we obtain H_{DMI}, as illustrated in Figure S2g for one case. The DMI constant is then calculated from the above expression for H_{DMI}. The observed zero velocity of DW (stationary DW) for fields near H_{DMI} comes from extrinsic pinning of the DWs in the racetrack (Ref. S2).

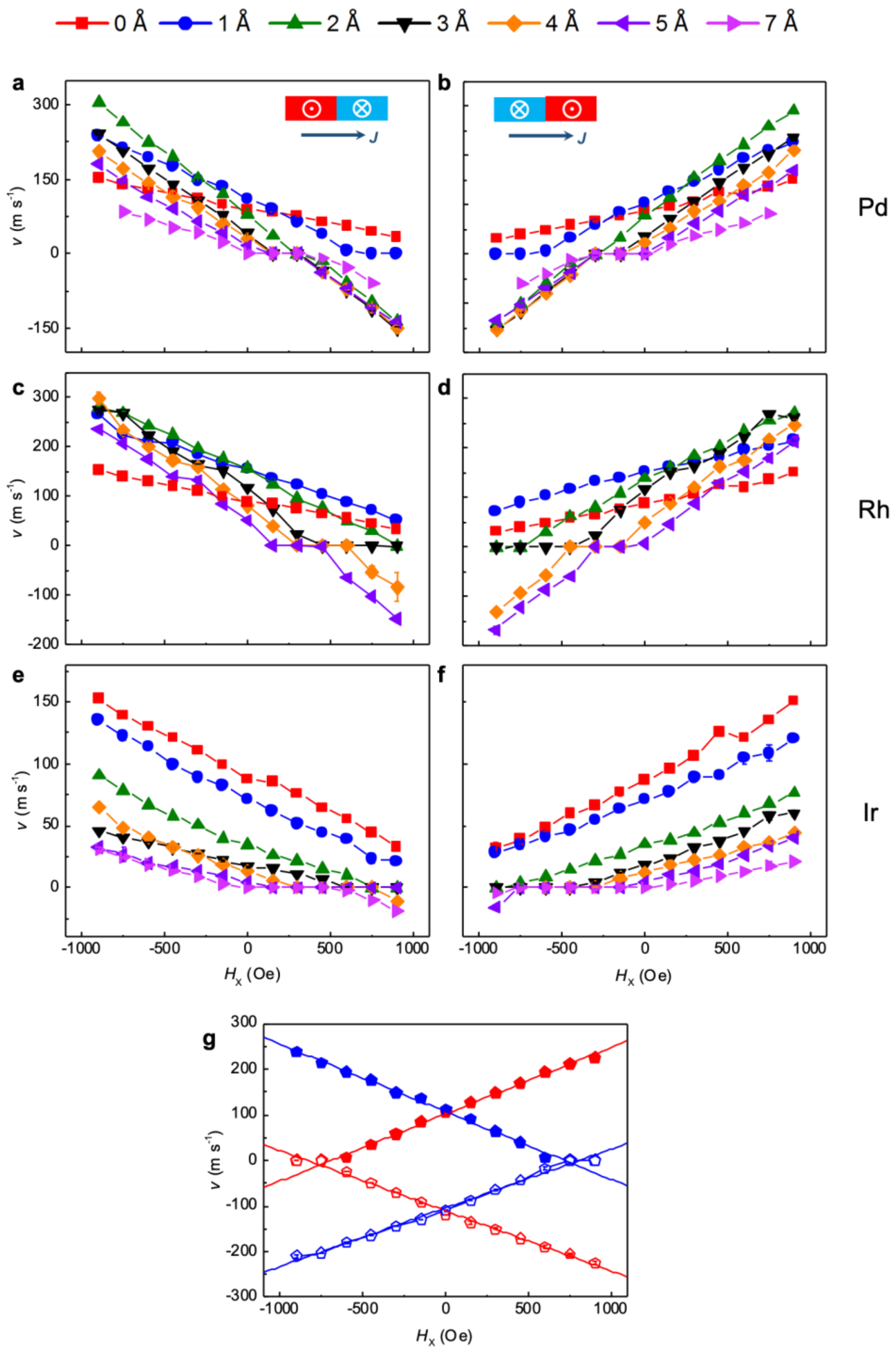

Figure S2 Experimental results of the dependence of the CIDWM on the longitudinal field, H_x, in all FM films with DLs. The current density is 1.2×10^8 A/cm^2 in all cases. Left and right panels show the up/down (↑↓) and down/up (↓↑) DW configurations, respectively. a) and b) correspond to a Pd DL, c) and d) to a Rh DL, and e) and f) to an Ir DL. DL thicknesses are indicated by: 0 Å - red squares, 1 Å - blue circles, 2 Å - olive triangles, 3 Å - black triangles, 4 Å - orange diamonds, 5 Å - purple triangles and 7 Å - violet triangles. The data for the 7 Å Pd DL is limited because of the smaller our of plane magnetic anisotropy field. No CIDWM was observed for the 7 Å Rh DL. Typical fitting of the data to extract the DMI field is shown in (g): the filled and open symbols correspond to positive and negative current injection, respectively, while blue and red represent the up/down and down/up domain configurations.

Section 3: 1-D model for the SAF structure

The 1-D model is extended to the SAF case based on the LLG formulism. The magnetization in each of the two AF coupled magnetic sub-layers $\boldsymbol{M}_i$, i = U (upper), L (lower), can be described as follows[S1, S2]:

$$\frac{\partial \boldsymbol{M}_i}{\partial t} = -\gamma \boldsymbol{M}_i \times \boldsymbol{H}_i^{\mathrm{eff}} + \frac{\alpha_i}{m_i} \boldsymbol{M}_i \times \frac{\partial \boldsymbol{M}_i}{\partial t} - b_{\mathrm{J}}^i \boldsymbol{M}_i \times \boldsymbol{M}_i \times \frac{\partial \boldsymbol{M}_i}{\partial t} - \beta_i b_{\mathrm{J}}^i \boldsymbol{M}_i \times \frac{\partial \boldsymbol{M}_i}{\partial x} + H_{\mathrm{she}}^i \boldsymbol{M}_i \times \boldsymbol{M}_i \times \hat{y}$$

where all the parameters have the same definition as in the previous section. In addition to the distinct magnetic parameters of the two sub-layers, the effective SHE field H_{SHE} is also different, because the spin current generated from the bottom Pt layer together with the DL will experience spin depolarization, as it diffuses through the lower FM layer, and finally, across the Ru spacer layer. Thus, we can write:

$$H_{\mathrm{SHE}}^U = H_{\mathrm{SHE}}^L \exp(-\frac{t_L}{\lambda_L} - \frac{t_{\mathrm{Ru}}}{\lambda_{\mathrm{Ru}}})$$

Here, λ_{Ru} is the spin diffusion length within the Ru spacer layer, and λ_L is the spin decoherence length of the lower FM layer. t_{L} is the thickness of the lower magnetic sub-layer and t_{Ru} is the thickness of the Ru layer. Here, we assume the DL together with the HM layer are the source for SOT generation.

We now focus on the effective field exerted on the system from the relationship $\boldsymbol{H}_i^{\mathrm{eff}} = \frac{\partial E}{\partial \boldsymbol{M}_i}$, where E is the the energy per unit area, which can be written as:

$$\begin{aligned} E = {} & 2A_L t_L \frac{\sin^2\theta_L}{\Delta^2} + t_L\left(K_L^{\mathrm{eff}} + K_L^u \cos^2\psi_L\right)\sin^2\theta_L - M_L H_L^{lg} \cos\psi_L \sin\theta_L + 2A_U t_U \frac{\sin^2\theta_U}{\Delta^2} \\ & + t_U\left(K_U^{\mathrm{eff}} + K_U^u \cos^2\psi_U\right)\sin^2\theta_U - M_U H_U^{lg} \cos\psi_U \sin\theta_U \\ & - 2J_{\mathrm{ex}}[\sin\theta_L \sin\theta_U \cos(\psi_L - \psi_U) + \cos\theta_L \cos\theta_U] \end{aligned}$$

Here, we assume an identical DW width in both the upper and lower magnetic layers as a result of the strong coupling between the two ferromagnetic sub-layers. K_i^u is the DW shape anisotropy constant, K_i^{eff} is the effective uniaxial anisotropy energy and H_i^{lg} is the total field in the longitudinal direction including the DMI effective field. The last term in the equation above is the exchange energy. These individual terms in E correspond to the various torques exerted on the ferromagnetic layers. The additional exchange term will give rise to an

exchange coupling torque which increases the DW velocity in the SAF structure significantly, as found in earlier work (Ref. S1).

By including these various effective fields into the LLG equation, we derive the following set of equations describing the DW motion in a SAF system:

$$\dot{q} = \frac{\alpha_L \alpha_U}{\alpha_U M_L(1+\alpha_L^2) + \alpha_L M_U(1+\alpha_U^2)}\left[-M_L\left(\frac{1}{\alpha_L}+\beta_L\right)u_L - M_U\left(\frac{1}{\alpha_U}+\beta_U\right)u_U \right.$$
$$\mp \frac{\gamma\Delta M_L}{\alpha_L}\{\frac{H_L^k}{2}\sin 2\psi_L - \frac{\pi}{2}H_L^{\mathrm{lg}}\sin\psi_L - \frac{2J_{\mathrm{ex}}}{M_L}\sin(\psi_L-\psi_U) + \frac{\alpha_L\pi H_L^{SH}}{2}\cos\psi_L\}$$
$$\pm \frac{\gamma\Delta M_U}{\alpha_U}\left\{\frac{H_U^k}{2}\sin 2\psi_U - \frac{\pi}{2}H_U^{\mathrm{lg}}\sin\psi_U - \frac{2J_{\mathrm{ex}}}{M_U}\sin(\psi_U-\psi_L)\right.$$
$$\left.\left. + \frac{\alpha_U\pi H_U^{SH}}{2}\cos\psi_U\right\}\right]$$

$$\dot{\psi}_L = \pm\frac{1}{\Delta}\frac{\alpha_U}{\alpha_U M_L(1+\alpha_L^2) + \alpha_L M_U(1+\alpha_U^2)}\left[-M_L\left(\frac{1}{\alpha_L}+\beta_L\right)u_L - M_U\left(\frac{1}{\alpha_U}+\beta_U\right)u_U \right.$$
$$\mp \frac{\gamma\Delta M_L}{\alpha_L}\left\{\frac{H_L^k}{2}\sin 2\psi_L - \frac{\pi}{2}H_L^{\mathrm{lg}}\sin\psi_L - \frac{2J_{\mathrm{ex}}}{M_L}\sin(\psi_L-\psi_U) + \frac{\alpha_L\pi H_L^{SH}}{2}\cos\psi_L\right\}$$
$$\pm \frac{\gamma\Delta M_U}{\alpha_U}\left\{\frac{H_U^k}{2}\sin 2\psi_U - \frac{\pi}{2}H_U^{\mathrm{lg}}\sin\psi_U - \frac{2J_{\mathrm{ex}}}{M_U}\sin(\psi_U-\psi_L)\right.$$
$$\left.\left. + \frac{\alpha_U\pi H_U^{SH}}{2}\cos\psi_U\right\}\right] + \frac{\gamma}{\alpha_L}\{\frac{H_L^k}{2}\sin 2\psi_L - \frac{\pi}{2}H_L^{\mathrm{lg}}\sin\psi_L - \frac{2J_{\mathrm{ex}}}{M_L}\sin(\psi_L-\psi_U)\}$$
$$\pm \frac{u_L}{\alpha_L\Delta}$$

$$\dot{\psi}_U = \mp\frac{1}{\Delta}\frac{\alpha_L}{\alpha_U M_L(1+\alpha_L^2) + \alpha_L M_U(1+\alpha_U^2)}\left[-M_L\left(\frac{1}{\alpha_L}+\beta_L\right)u_L - M_U\left(\frac{1}{\alpha_U}+\beta_U\right)u_U \right.$$
$$\mp \frac{\gamma\Delta M_L}{\alpha_L}\left\{\frac{H_L^k}{2}\sin 2\psi_L - \frac{\pi}{2}H_L^{\mathrm{lg}}\sin\psi_L - \frac{2J_{\mathrm{ex}}}{M_L}\sin(\psi_L-\psi_U) + \frac{\alpha_L\pi H_L^{SH}}{2}\cos\psi_L\right\}$$
$$\pm \frac{\gamma\Delta M_U}{\alpha_U}\left\{\frac{H_U^k}{2}\sin 2\psi_U - \frac{\pi}{2}H_U^{\mathrm{lg}}\sin\psi_U - \frac{2J_{\mathrm{ex}}}{M_U}\sin(\psi_U-\psi_L)\right.$$
$$\left.\left. + \frac{\alpha_U\pi H_U^{SH}}{2}\cos\psi_U\right\}\right] + \frac{\gamma}{\alpha_U}\{\frac{H_U^k}{2}\sin 2\psi_U - \frac{\pi}{2}H_U^{\mathrm{lg}}\sin\psi_U - \frac{2J_{\mathrm{ex}}}{M_U}\sin(\psi_U-\psi_L)\}$$
$$\pm \frac{u_U}{\alpha_U\Delta}$$

Where all the parameters have the same definition as the FM case, but with the subscript corresponding to the upper (U) and lower (L) sub-layers. By deriving the steady state solution that corresponds to $\dot{\psi}_L = \dot{\psi}_U = 0$, we can calculate the longitudinal field dependence of the DW velocity in samples with DLs. Figure S3 gives the longitudinal field dependence of the

CIDWM in all SAF films with DLs. Figure S4-S9 compare the measured v-H_x curves with the fitted curves. All the fitting parameters are sample specific except for the antiferromagnetic exchange coupling which is determined only by the Ru layer thickness, since it depends on the electronic properties of the Ru itself plus the interfacial exchange between the Ru layer and the adjacent magnetic Co layers.

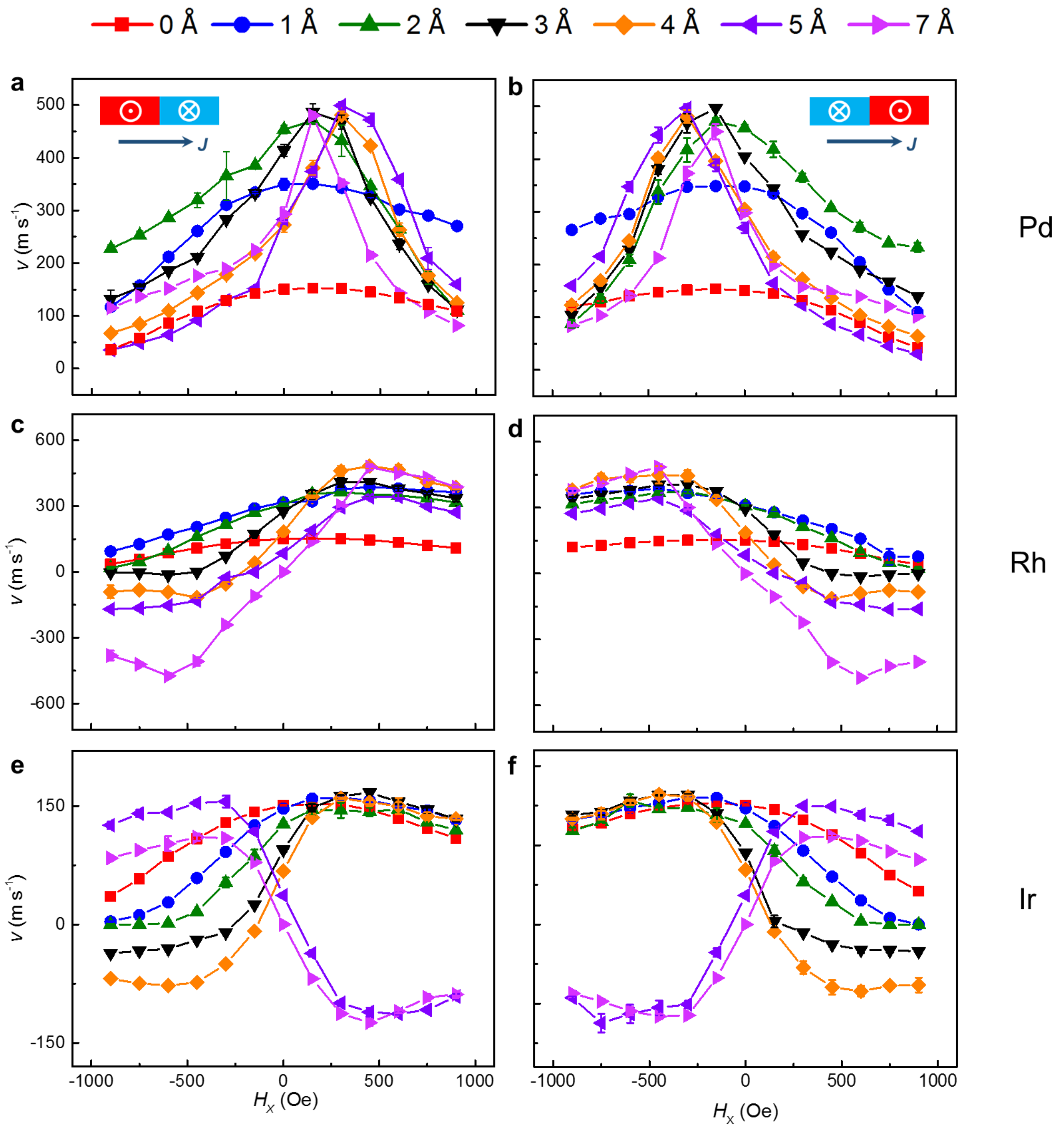

Figure S3 Experimental results of longitudinal field dependence of the CIDWM in all SAF samples with DLs. The current density in the measurements is fixed at 1.2×10^8 A/cm^2. Left and right panels show the up/down (↑↓) and down/up (↓↑) domain wall configurations, respectively. a) and b) correspond to the Pd DL case, c) and d) to the Rh DL case and e) and f) to the Ir DL case. DL thicknesses are indicated as follows: 0 Å - red squares, 1 Å - blue circles, 2 Å - olive triangles, 3 Å - black triangles, 4 Å - orange diamonds, 5 Å - purple triangles and 7 Å - violet triangles.

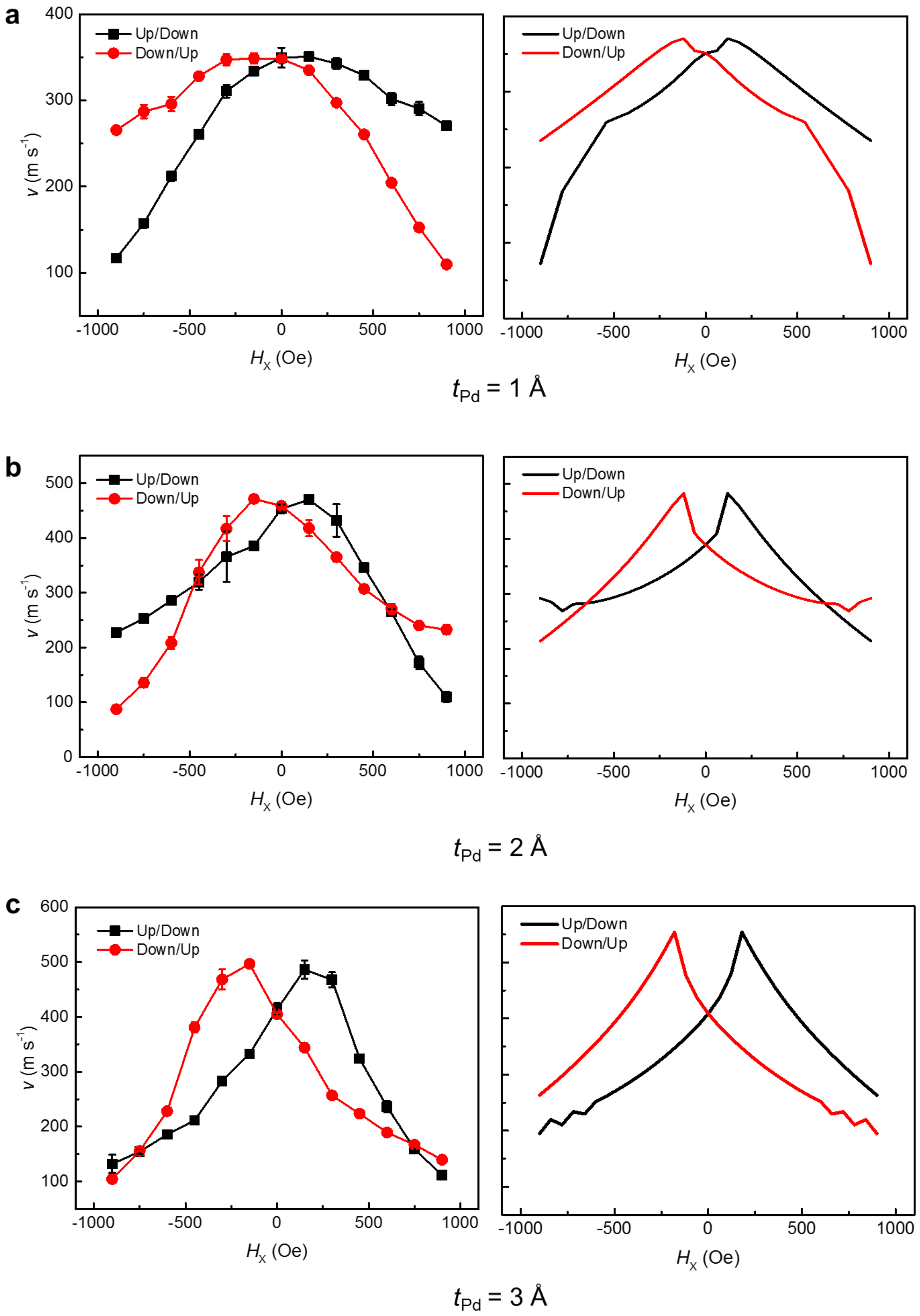

Figure S4 Experimental results of longitudinal field dependence of the DW velocity in the Pd DL system (left panel) and the corresponding simulations (right panel) based on the 1-D model with DL thicknesses of: 1 Å (a), 2 Å (b) and 3 Å (c).

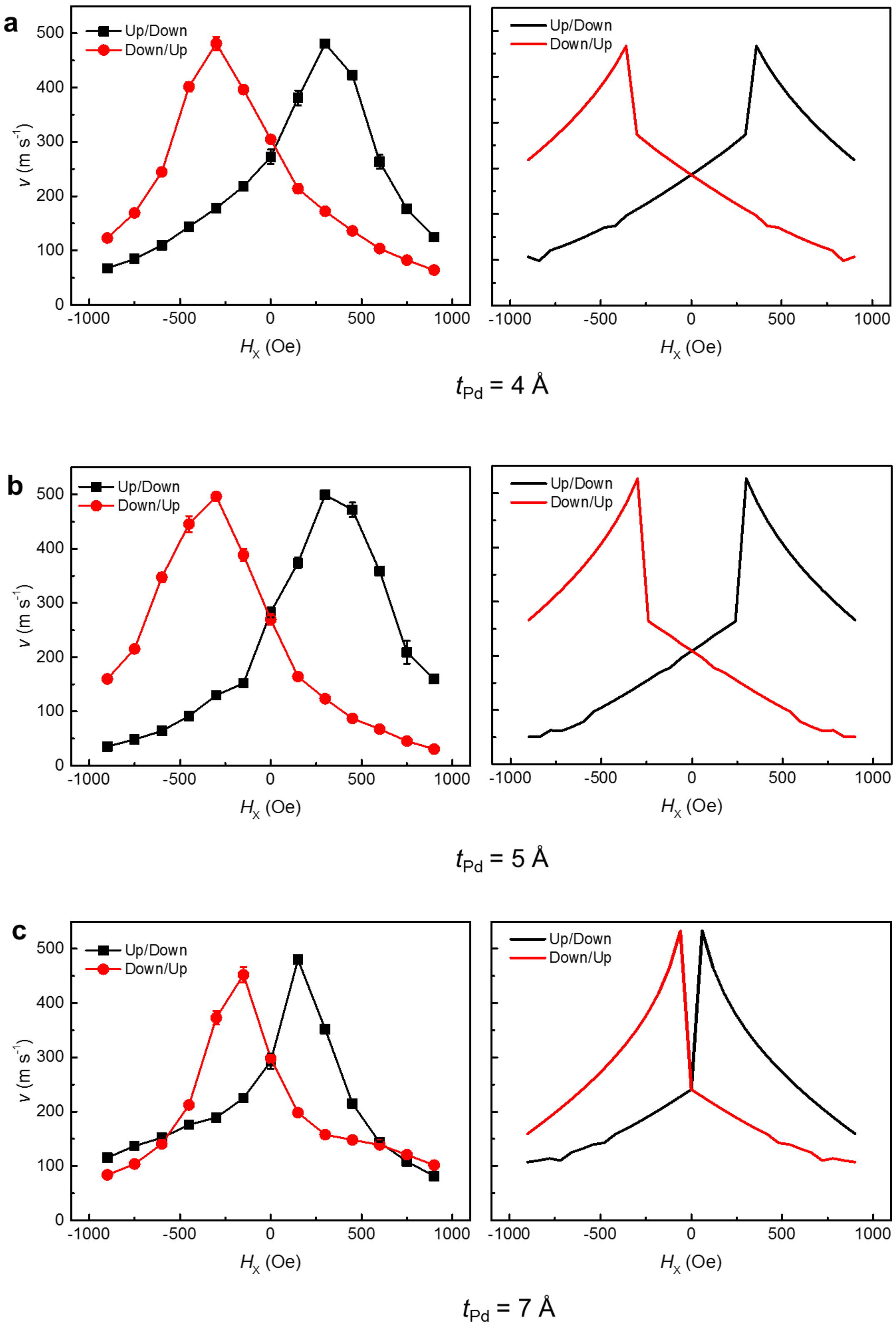

Figure S5 Experimental results of longitudinal field dependence of the DW velocity in the Pd DL system (left panel) and the corresponding simulations (right panel) based on the 1-D model with DL thicknesses of: 4 Å (a), 5 Å (b) and 7 Å (c).

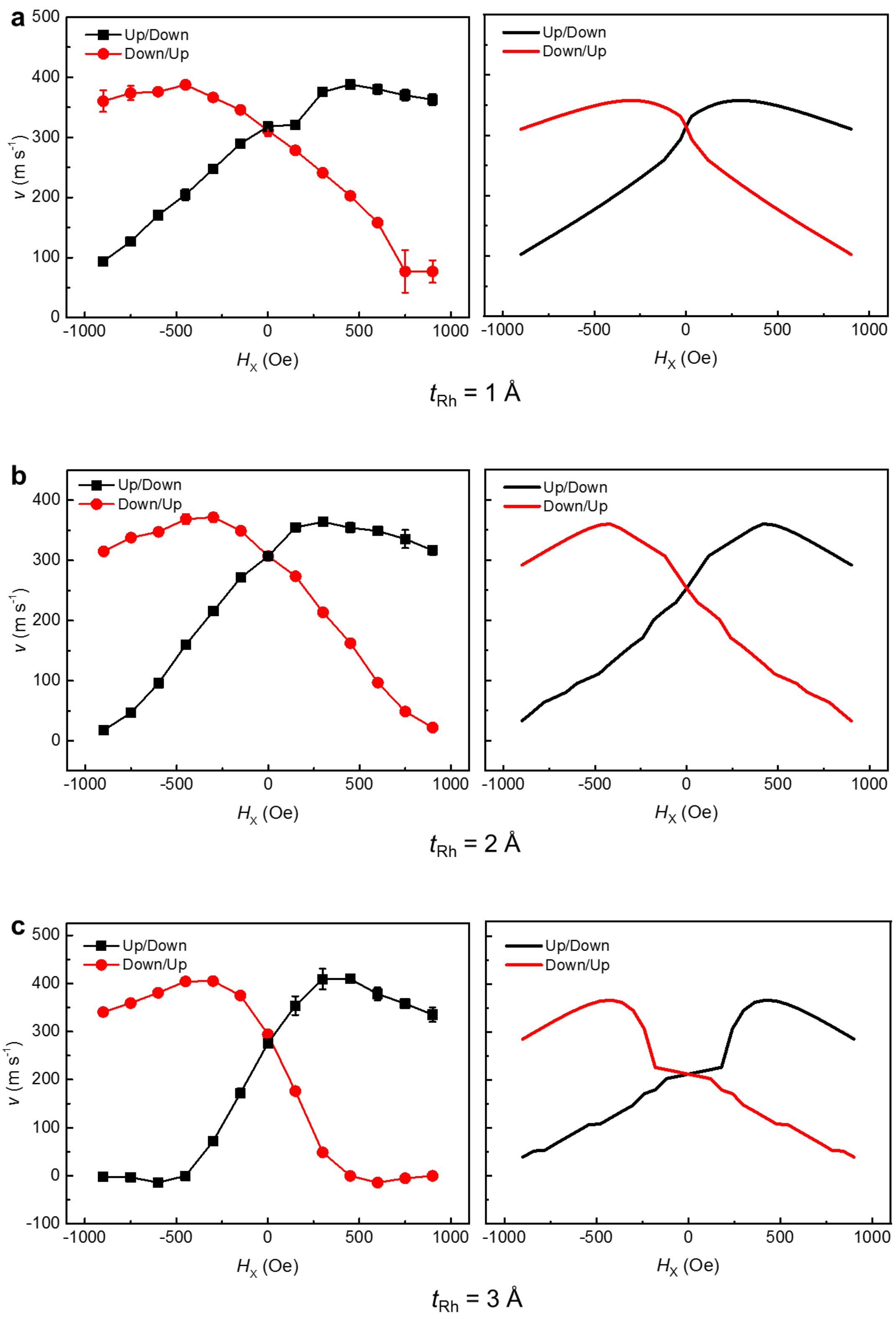

Figure S6 Experimental results of longitudinal field dependence of the DW velocity in the Rh DL system (left panel) and the corresponding simulations (right panel) based on the 1-D model with DL thicknesses of: 1 Å (a), 2 Å (b) and 3 Å (c).

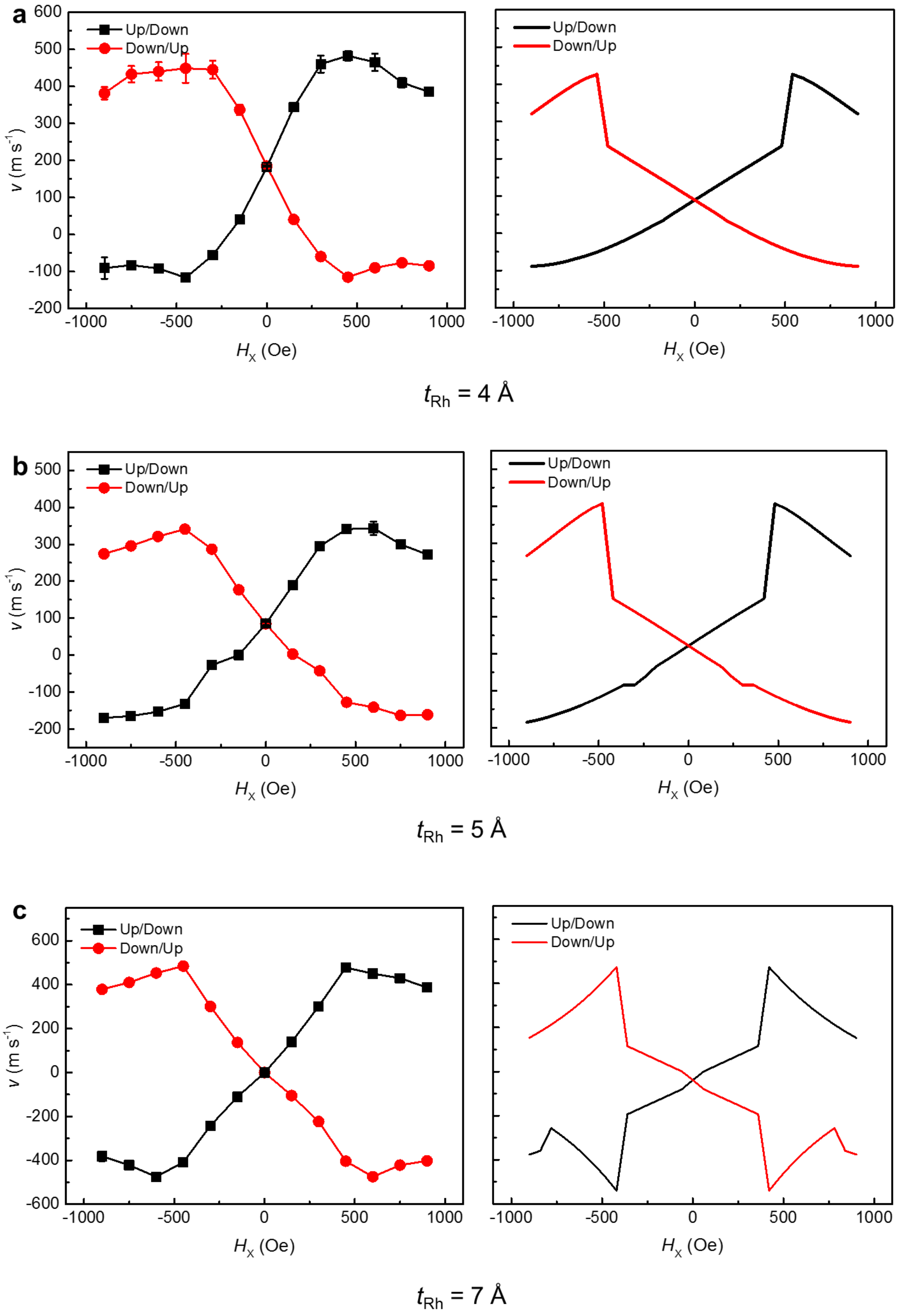

Figure S7 Experimental results of longitudinal field dependence of the DW velocity in the Rh DL system (left panel) and the corresponding simulations (right panel) based on the 1-D model with DL thicknesses of: 4 Å (a), 5 Å (b) and 7 Å (c).

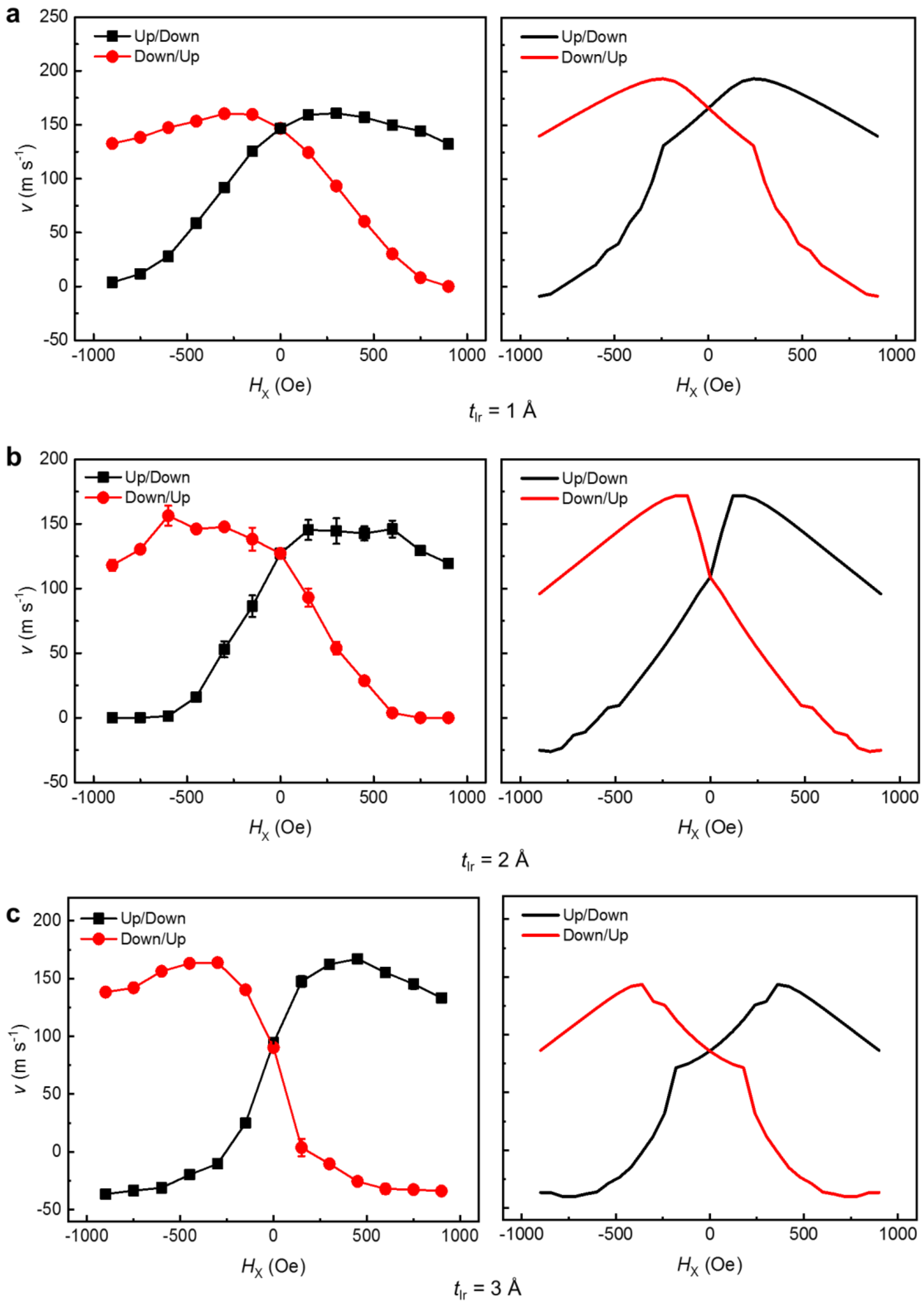

Figure S8 Experimental results of longitudinal field dependence of the DW velocity in the Ir DL system (left panel) and the corresponding simulations (right panel) based on the 1-D model with DL thicknesses of: 1 Å (a), 2 Å (b) and 3 Å (c).

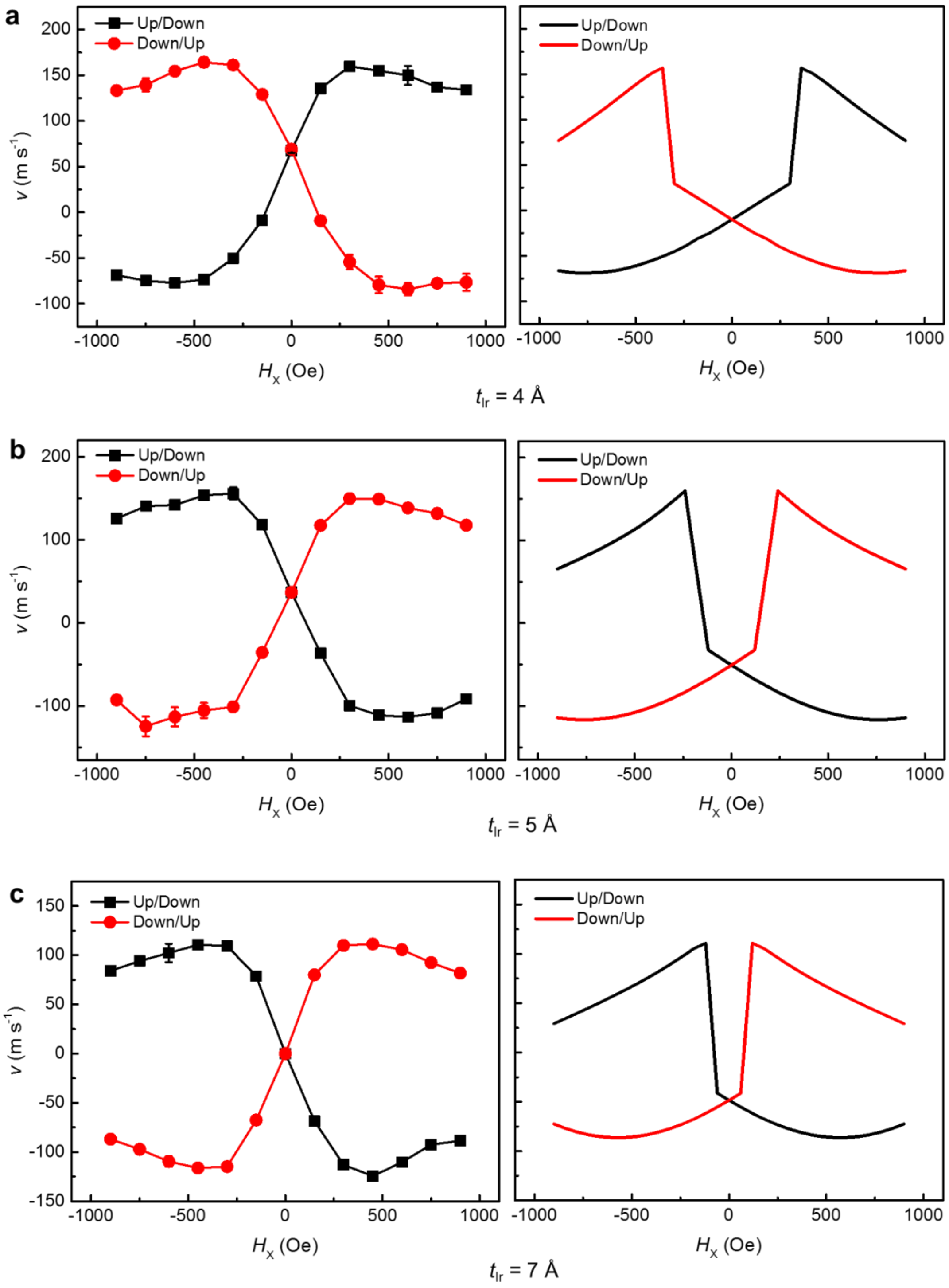

Figure S9 Experimental results of longitudinal field dependence of the DW velocity in the Ir DL system (left panel) and the corresponding simulations (right panel) based on the 1-D model with DL thicknesses of: 4 Å (a), 5 Å (b) and 7 Å (c).

Section 4. Illustration of v_{lg} by simulations from the 1-D analytical model and deduction of ECT-dominated CIDWM

In this section we discuss the physical meaning of v_{lg} that was introduced in the main text (Figure 5g). From the 1-D analytical model for CIDWM in a SAF system, the DW velocity $\dot{q}$ can be written, under steady-state conditions, as:

$$\dot{q} = \frac{1}{\alpha_U M_L + \alpha_L M_U}\left[-M_L\beta_L u_L - M_U\beta_U u_U \mp \frac{\gamma\Delta M_L \pi H_L^{\text{SH}}}{2}\cos\psi_L \pm \frac{\gamma\Delta M_U \pi H_U^{\text{SH}}}{2}\cos\psi_U\right]$$

$$\dot{q} = \mp\gamma\Delta[\frac{H_L^k}{2}\sin 2\psi_L - \frac{\pi}{2}H_L^{\text{lg}}\sin\psi_L - \frac{2J_{\text{ex}}}{M_L}\sin(\psi_L - \psi_U)] \mp u_L$$

$$\dot{q} = \pm\gamma\Delta[\frac{H_U^k}{2}\sin 2\psi_U - \frac{\pi}{2}H_U^{\text{lg}}\sin\psi_U - \frac{2J_{\text{ex}}}{M_U}\sin(\psi_U - \psi_L)] \mp u_U$$

where α_i is the damping parameter of each sublayer, with i corresponding to the L (lower) or U (upper) layer; M_i is the magnetization; β_i is the non-adiabatic constant; u_i is the STT-related (spin-transfer torque) DW velocity; γ is the gyromagnetic ratio; Δ is the DW width; H_i^k is the in-plane shape anisotropy field that favors a Bloch wall; H_i^{lg} is the net longitudinal magnetic field that includes H_x, the applied field, and the DMI effective field; ψ_i is the angle between the inner magnetization direction of the DW in each layer and the x-axis; H_i^{SH} is the spin Hall effective field in each layer; J_{ex} is the interlayer exchange coupling constant.

When an exterior longitudinal field is applied to the system, as indicated from the first equation above, the velocity peaks at the field where the SOTs are maximized at $\psi_L = 0$ or π since the lower layer experiences a larger SOT than the upper layer (see Section 4 in SI and Figure S11 for the simulation results). When $M_L = M_U = M$ and neglecting the STT-related terms, and by considering the case when $\psi_L = 0$ or π, then the set of equations above can be rewritten in the following form:

$$v_{\text{lg}} = \frac{\pi\gamma\Delta}{2\alpha}[\pm\frac{H_L^{\text{SH}}}{2} \pm \frac{H_U^{\text{SH}}}{2}\cos\psi_U]$$

$$v_{\text{lg}} = \pm\frac{2\gamma\Delta J_{\text{ex}}}{M}\sin\psi_U$$

$$\cos\psi_U = \frac{\pi H_{\text{lg}}^U}{2H_U^k}$$

Thus, v_{lg} directly reflects the magnitude of SOT exerted on both the lower and upper layers.

We further utilize the 1-D analytical model for simulating the longitudinal field dependence of the SAF CIDWM. The simulation parameters used are shown in Table S1. From the simulation results in Figure S10, as the current density is increased, the spin Hall effective field and STT-related velocity also increase, so that the peak DW velocity v_{lg} is located at the point where the magnetization of the lower layer is oriented at an angle ψ_L~π for an up-down DW configuration (both the spin Hall effective field used in the simulations and in the experiments are very small compared to the exchange coupling field). (Note that for the up-down DW configuration, ψ_L ~0). This is understandable since at this DW magnetization angle, the DW velocity contributed by the SOT is maximized in the lower layer (which has a larger SOT compared to the upper layer). Indeed, with increased current density, the peak position of the v-H_x curve becomes closer to zero field. When the current becomes even larger so that the velocity derived from SOT is comparable to $\gamma\Delta\frac{2J_{\mathrm{ex}}}{M}$, the above assertion that the value of ψ_L is ~π (or 0) is longer true, and v_{lg} will no longer be realized at $\psi_L = 0$ or π but emerge at zero-field. Also, note that the DW velocity contribution from the ECT $(-\frac{2J_{\mathrm{ex}}}{M_L}\sin(\psi_L - \psi_U))$ is always dominant compared to the DMI-related contribution $(\frac{H_L^k}{2}\sin 2\psi_L - \frac{\pi}{2}H_L^D\sin\psi_L)$ and the exterior longitudinal field contribution $(-\frac{\pi}{2}H_L^{\mathrm{lg}}\sin\psi_L)$. Here we discuss two different conditions to give insight into the ECT-driven CIDWM.

1. Small exchange coupling:

We now give an illustration of the v-J curve for an ECT dominated case, when the two magnetic sub-layers are similar to each other ($M_L = M_U = M \& \alpha_L = \alpha_U = \alpha$). The equations now have the following form:

$$\dot{q} = \frac{1}{2\alpha}\left[\mp\frac{\gamma\Delta\pi H_L^{SH}}{2}\cos\psi_L \pm \frac{\gamma\Delta\pi H_U^{SH}}{2}\cos\psi_U\right]$$

$$\dot{q} = \mp\gamma\Delta\{-\frac{2J_{\mathrm{ex}}}{M}\sin(\psi_L - \psi_U)\}$$

$$\dot{q} = \pm\gamma\Delta\{-\frac{2J_{\mathrm{ex}}}{M}\sin(\psi_U - \psi_L)\}$$

By expanding the second equation, we obtain:

$$\dot{q} = \mp\gamma\Delta\frac{2J_{\mathrm{ex}}}{M}\{-\sin\psi_L\cos\psi_U + \sin\psi_U\cos\psi_L\}$$

When the DW moves, the magnetization in each sub-layer can take arbitrary angles. Thus, we obtain the following relationships between the angles of the magnetizations of the two sub-layers and the spin Hall angle fields:

$$H_L^{SH} = \frac{8\alpha J_{\text{ex}}}{\pi M}\sin\psi_U$$

$$H_U^{SH} = \frac{8\alpha J_{\text{ex}}}{\pi M}\sin\psi_L$$

By inserting these relationships into the equations above, we obtain the DW velocity in the following form:

$$\dot{q} = \pm\frac{\pi\gamma\Delta}{4\alpha}[H_L^{SH}\sqrt{1-(\frac{H_U^{SH}}{H_{\text{ex}}})^2} + H_U^{SH}\sqrt{1-(\frac{H_L^{SH}}{H_{\text{ex}}})^2}]$$

where $H_{\text{ex}} = \frac{8\alpha J_{\text{ex}}}{\pi M}$ is defined as the exchange coupling torque field. From the above derivations, we conclude that: the DW velocity is proportional to $J\sqrt{1-(\frac{J}{J_{\text{ECT}}})^2}$ (where $J_{\text{ECT}} = \frac{16et\alpha J_{\text{ex}}}{\pi\hbar\theta_{\text{SH}}}$) and has a maximum value of $\frac{2\gamma\Delta J_{\text{ex}}}{M}$ when the magnetizations in the upper and lower DWs are perpendicular to each other. As the current is increased, the DW velocity will quickly drop and goes to zero when $H_L^{SH} = \frac{8\alpha J_{\text{ex}}}{\pi M}$. One thing worth noting is that when the current density is low, the velocity is approximately linearly proportional to *J* with a fixed slope which depends only on the SOT term. The comparison of *v*-*J* curves in a DMI-dominated model and ECT-dominated model is shown in Figure S10j. In the low current density region as $J \ll J_{\text{ECT}}$, we can derive the DW velocity as $\dot{q} = \pm\frac{\pi\gamma\Delta}{4\alpha}[H_L^{SH} + H_U^{SH}]$, so the DW mobility around this region will be proportional to the net SOT exerted on the whole system.

2. Large exchange coupling

We can also derive the following equations that describe the current induced motion with an extremely large J_{ex}. The equations become:

$$\dot{q} = \frac{1}{\alpha_U M_L + \alpha_L M_U}\left[-M_L\beta_L u_L - M_U\beta_U u_U \mp \frac{\gamma\Delta M_L \pi H_L^{SH}}{2}\cos\psi_L \pm \frac{\gamma\Delta M_U \pi H_U^{SH}}{2}\cos\psi_U\right]$$

$$\dot{q} = \mp\gamma\Delta\{\frac{H_L^k}{2}\sin2\psi_L - \frac{\pi}{2}H_L^{\mathrm{lg}}\sin\psi_L - \frac{2J_{\mathrm{ex}}}{M_L}\sin(\psi_L - \psi_U)\} \mp u_L$$

$$\dot{q} = \pm\gamma\Delta\{\frac{H_U^k}{2}\sin2\psi_U - \frac{\pi}{2}H_U^{\mathrm{lg}}\sin\psi_U - \frac{2J_{\mathrm{ex}}}{M_U}\sin(\psi_U - \psi_L)\} \mp u_U$$

By ignoring the STT-term and focusing on the remnant magnetization $M_r = M_L - M_U$, we find the following expressions:

$$\dot{q} = \frac{M_r}{\alpha_U M_L + \alpha_L M_U}\left[\mp\frac{\gamma\Delta M_L \pi H_L^{SH}}{2M_r}\cos\psi_L \pm \frac{\gamma\Delta M_U \pi H_U^{SH}}{2M_r}\cos\psi_U\right]$$

$$\dot{q} = \mp\gamma\Delta\{\frac{M_L H_L^k}{2M_r}\sin2\psi_L - \frac{\pi M_L H_L^{\mathrm{lg}}}{2M_r}\sin\psi_L + \frac{M_U H_L^k}{2M_r}\sin2\psi_U - \frac{\pi M_U H_U^{\mathrm{lg}}}{2M_r}\sin\psi_U\}$$

For the strong exchange coupling case, the magnetization of the upper and lower DWs will form an angle close to π, which yields $\cos\psi_U \sim -\cos\psi_L$ and $\sin\psi_U \sim -\sin\psi_L$, so that the above equations can be rewritten as follows:

$$\dot{q} = \mp\frac{M_r}{\alpha_U M_L + \alpha_L M_U}\left[\frac{\gamma\Delta M_L \pi H_L^{SH}}{2M_r} + \frac{\gamma\Delta M_U \pi H_U^{SH}}{2M_r}\right]\cos\psi_L$$

$$\dot{q} = \mp\gamma\Delta\{\frac{(M_L H_L^k + M_U H_U^k)}{2M_r}\sin2\psi_L - \frac{\pi(M_L H_L^{\mathrm{lg}} - M_U H_U^{\mathrm{lg}})}{2M_r}\sin\psi_L\}$$

It is obvious that these forms are exactly the same as the DW motion in the single ferromagnetic layer case with the presence of DMI but with some modified parameters, as follows:

$$\alpha_r = \frac{\alpha_U M_L + \alpha_L M_U}{M_r}$$

$$H_r^{SH} = \frac{M_L H_L^{SH} + M_U H_U^{SH}}{2M_r}$$

$$H_r^k = \frac{(M_L H_L^k + M_U H_U^k)}{2M_r}$$

$$H_r^{\mathrm{DMI}} = \frac{(M_L H_L^{\mathrm{DMI}} - M_U H_U^{\mathrm{DMI}})}{2M_r}$$

So, for the case of large exchange coupling, the CIDWM will indeed behave just like the single layer case. But one thing worth noticing is that, even though we have already made

the assumption that $\psi_U \sim \psi_L + \pi$ and have eliminated the ECT term by simple arithmetic for the coupled DW case, we find from the simulations, that we can still observe a considerable contribution of the ECT term even though the angle between the two DW magnetizations is only slightly deviated from π as shown in Figure S10i. In any case, even though the whole system displays very much a single layer like behavior, in each sub-layer the existence of the ECT is still the main reason for the observation of fast DW velocity.

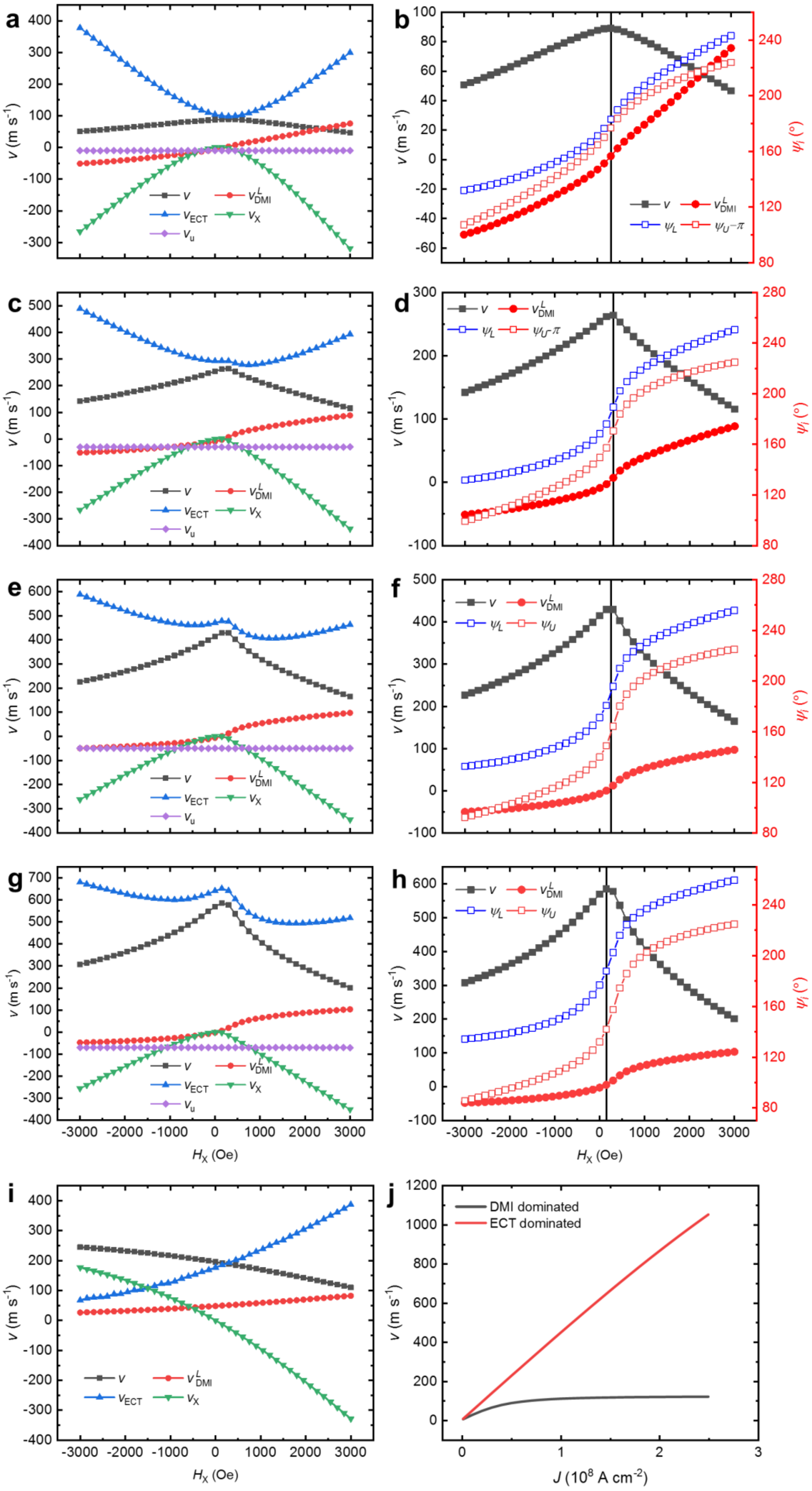

a
b
c
d
e
f
g
h
i
j
v (m s-1)
ψl (°)
HX (Oe)
J (10^8 A cm-2)
v
vECT
vu
vLDMI
vX
ψL
ψU-π
ψU
DMI dominated
ECT dominated

Figure S10 Simulation results for the dependence of the SAF CIDWM versus longitudinal field. a), c), e), g) and i), contributions to the DW velocity in the lower (or upper) magnetic layer from different torques: total velocity (black square), DMI-related velocity (red circle), ECT-origin velocity (blue triangle), longitudinal-field induced velocity (green triangle) and STT-term velocity (violet diamond). b), d), f) and h), the response of SAF DW velocity (black square), DMI-related velocity (red circle) and DW magnetization angle of the upper (red unfilled square) and lower (blue unfilled square) layer to the longitudinal field. j), illustrations of the *v*-*J* curve in 1-D DMI-dominated (black curve) and ECT-dominated (red curve) models. The simulation parameters are summarized in Table S1.

Table S1 Summary of parameters used for the simulation results presented in Figure S10a-i in Section 4.

	**Figure S10**	**(a)** and **(b)**	**(c)** and **(d)**	**(e)** and **(f)**	**(g)** and **(h)**	**(i)**
1-D model simulation	Δ (nm)	4.3	4.3	4.3	4.3	4.3
	u (m/s)	10	30	50	70	30
	α_L	0.1	0.1	0.1	0.1	0.1
	α_U	0.1	0.1	0.1	0.1	0.1
	β_L	0	0	0	0	0
	β_U	0	0	0	0	0
	H_L^k (Oe)	1000	1000	1000	1000	1000
	H_U^k (Oe)	1000	1000	1000	1000	1000
	M_L/M_U	1	1	1	1	0.8
	J_{ex} (erg/cm^2)	-0.3	-0.3	-0.3	-0.3	-12
	H_L^{SH} (Oe)	110	330	550	770	330
	H_U^{SH} (Oe)	40	120	200	280	120
	H_L^{DMI} (Oe)	-1000	-1000	-1000	-1000	-1000
	H_U^{DMI} (Oe)	400	400	400	400	400

Section 5: Enhancement of the thermal stability in the SAF case by the RKKY interaction

The thermal stability of a spintronic device is critical in real world applications. In the FM case, the thermal stability is determined by the effective uniaxial anisotropy K_u^{eff}. Defining the thermal stability coefficient $\tau = K_u^{\text{eff}} V/k_B T$, for a device with a size of 100nm×100nm×2nm, and for K_u^{eff}~1Merg/cm^3, this will give τ ~500, which is extremely large and well exceeds what is needed for real-world applications. However, in the SAF case, the thermal stability is further enhanced through the coupling between the two magnetic sub-layers. As derived from Section 3, the energy density per unit area under the application of an out-of-plane magnetic field can be written as:

$$E = -t_L K_L^{\text{eff}} \cos^2\theta_L - t_U K_U^{\text{eff}} \cos^2\theta_U - H_Z t_U M_U \cos\theta_U - H_Z t_L M_L \cos\theta_L - 2J_{\text{ex}}[\sin\theta_L \sin\theta_U \cos(\psi_L - \psi_U) + \cos\theta_L \cos\theta_U]$$

For simplicity, we take two identical sub-layers, which means $t_L = t_U = t$, $K_U^{\text{eff}} = K_L^{\text{eff}} = K^{\text{eff}}$ and $M_L = M_U = M$, and we consider $\psi_L = \psi_U$. The energy density term is then simplified as:

$$E = -tK^{\text{eff}}(\cos^2\theta_L + \cos^2\theta_U) - tH_Z M(\cos\theta_U + \cos\theta_L) - 2J_{\text{ex}}[\cos(\theta_U - \theta_L)]$$

By comparison with the FM case, the energy difference between the up and down state for each sub-layer is no longer K^{eff}, but $\sqrt{-K^{\text{eff}}(K^{\text{eff}} + 2J_{\text{ex}}/t)}$ for $K^{\text{eff}} < -J_{\text{ex}}/t$, as shown in Figure S11a. Thus, the thermal stability in the SAF case can be well maintained even though K^{eff} of each sub-layer is decreased. For example, for the case where $J_{\text{ex}} = -0.5$ erg/cm and $t = 1$nm, when the K^{eff} of each sub-layer decreases from 4 Merg/cm^3 to 2 Merg/cm^3, the thermal stability of the SAF only decreases from 4.9 Merg/cm^3 to 4 Merg/cm^3 as shown in Figure S11a.

The higher thermal stability of the DWs in a SAF structure is further manifested by examining the dependence of threshold current density J_{th} on the current pulse length and temperature. J_{th} in the FM case is more sensitive to both the pulse length and temperature than in the SAF case, independent of the DL (Figure S11b, c and d).

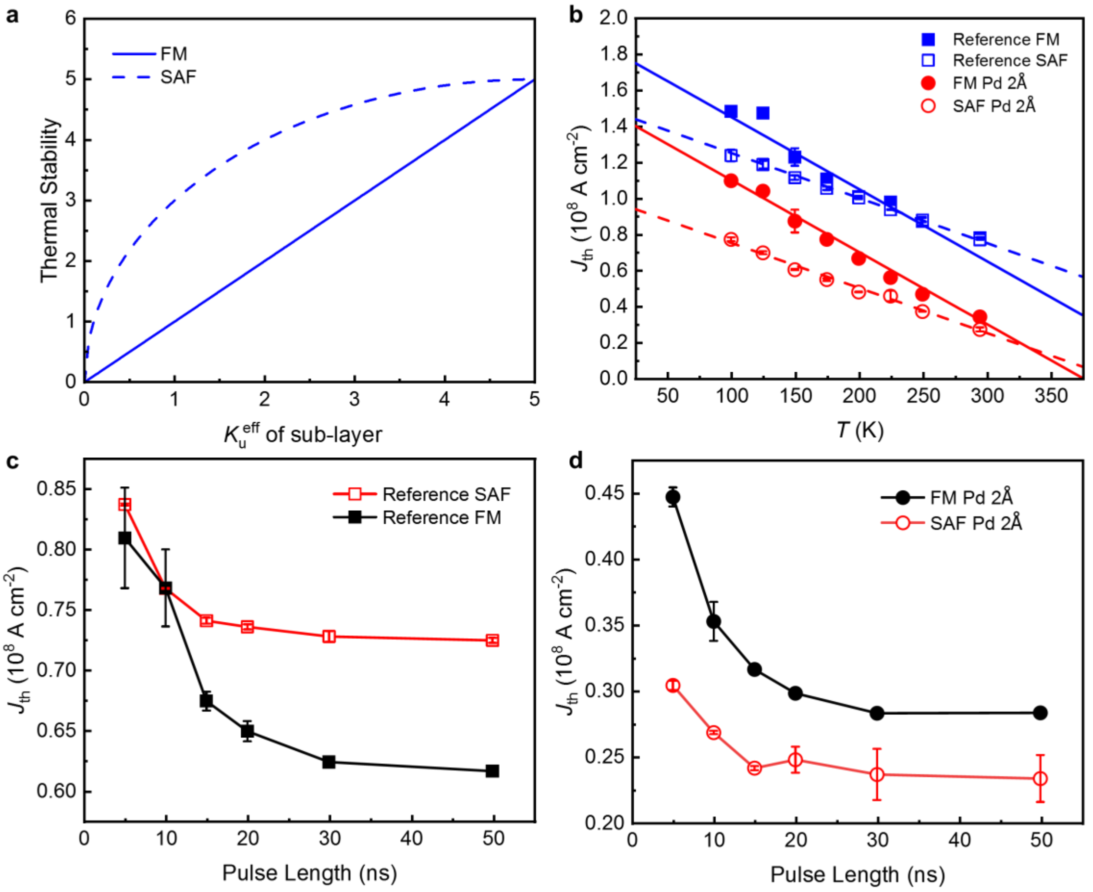

Figure S11 Thermal stability comparison of FM and SAF samples with/without DL insertions. a) The calculated thermal stability of FM and SAF structures as a function of the K_u^{eff} of the sub-layers. b) The temperature dependence of the threshold current density J_{th} in both the reference FM (blue filled square), reference SAF (blue unfilled square), FM with 2Å Pd DL (red filled circle) and SAF with 2Å Pd DL (red unfilled circle). The FM case shows a more sensitive response than the SAF case, as the slope of the lines are larger. The pulse length dependence of J_{th} in reference samples (c) and samples with 2Å Pd DL (d). In the FM case, J_{th} is more easily affected by the pulse length.

Section 6: Analysis of CIDWM curves subtracting the threshold current density

It has always been controversial as to why there is a threshold current density below which the DW does not move. Figure S12 exhibits the rescaled *v*-*J* curves by subtracting the threshold current density. In the FM case, by fitting the *v*-*J* curve at the current density region below 15×10^{11} A m^{-2} (avoiding thermal effects) with the following equation: $v = v_D/\sqrt{1+(\frac{J_D}{J})^2}$ (where $v_D = \pi\gamma_0 D/\mu_0 M_S$ and $J_D = 2\alpha t e D/(\hbar\theta_{SH}\Delta)$, Ref. S3, S5 and S6), we obtain both v_D and J_D. When the current density is small, such that $J \ll J_D$, we find $v = \frac{v_D}{J_D}J = \frac{\pi\gamma_0\hbar\theta_{SH}\Delta}{2M_S\alpha te}J$ so that the slope, which is the DW mobility, is given by, as discussed in the main text, $\xi_{DW} = \frac{\pi\gamma_0\hbar\theta_{SH}\Delta}{2M_S\alpha te}$. The mobility depends on the parameters θ_{SH}, Δ, α and M_S and, most interestingly, is independent of *D*. To double-check our analysis, we compare the value of $\frac{v_D}{J_D}$ obtained from fitting the rescaled experimental data with the 1-D model, with the value obtained from the CIDWM measurements (determined for current densities below 3×10^{11} A m^{-2}) as discussed in the main text. We find that these are in excellent agreement with one another, except for the thick Pd DL cases which display an ultra-small J_D (as shown in Figure S12g). We also compare the value of *D* calculated from results of fitting $v_D M_S/\pi\gamma_0$ with the values of *D* measured from the longitudinal field dependence: a good correspondence is observed, as shown in Figure S12h which further supports the reliability of our analysis.

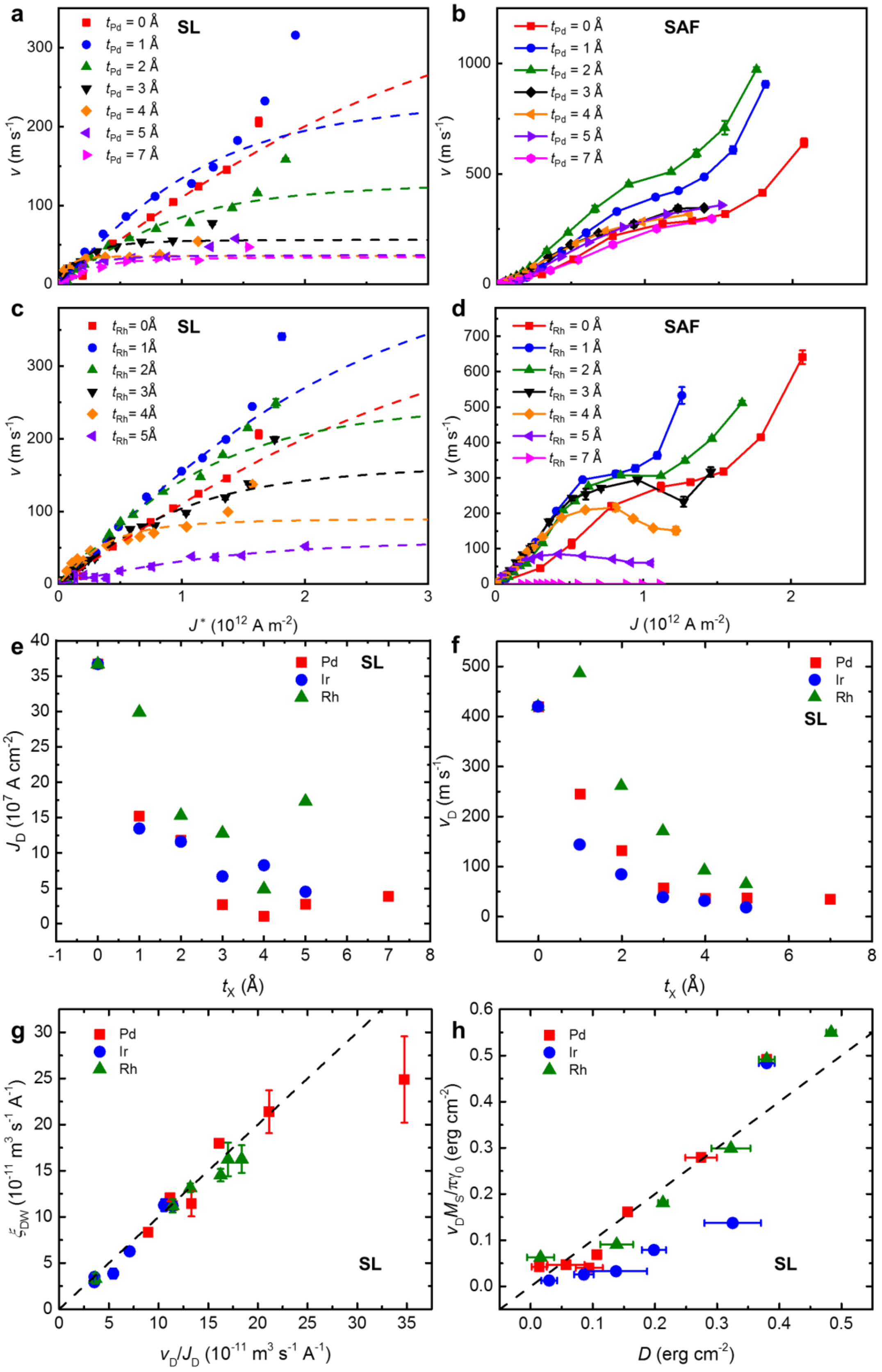

Figure S12 CIDWM curves rescaled by subtracting the threshold current density. The FM case with Pd DL (a) and Rh DL (c), SAF case with Pd DL (b) and Rh DL (d), the fitted lines

in (a) and (c) are following the equations of $v = v_D/\sqrt{1+(\frac{J_D}{J})^2}$. DL thicknesses are indicated as follows: 0 Å - red squares, 1 Å - blue circles, 2 Å - olive triangles, 3 Å - black triangles, 4 Å - orange diamonds, 5 Å - purple triangles and 7 Å - violet triangles. The DL thickness dependence of the fitted saturation current density J_D (e) and the DW velocity v_D (f) in the FM case are presented; the comparison between the DW mobility and v_D/J_D value from fitting in the FM case (g) and the D value calculated from $v_D M_S/\pi\gamma_0$ with value of D from the longitudinal field dependence (h). The dashed lines are lines across the original point with slope of 1. The Pd, Ir, and Rh DLs are represented by red squares, blue circles and olive triangles, respectively.

Section 7: Investigation of the effective spin Hall angle from the 1-D model of CIDWM in the FM case

We also investigate the effective spin Hall angle (θ_{SH}) based on the model in Section 6. From this derivation, we find that $\theta_{SH} \propto \frac{\alpha M_S \xi_{DW}}{\Delta}$. By plotting the normalized $\frac{\alpha M_S \xi_{DW}}{\Delta}$ versus the DL thickness, as shown in Figure S13, different behaviors are observed depending on the DL. For the Ir DL case, θ_{SH} shows a monotonic decrease with increasing Ir thickness; by contrast, for the Pd and Rh cases, θ_{SH} shows a modest increase initially for thin DLs but a decrease as the DL thickens.

Neither Pd or Ir (nor Rh that shows no PMA here) is a promising candidate for the HM layer in ref. [S2], which is consistent with the thick DL region as observed in our case. In this work, we have observed that these materials have distinct effects when they serve as atomically thin DLs. The Ir DL shows almost the same behavior as the Au DL case [ref. S2] with less efficient CIDWM since they are both 5*d* elements and negate the proximity induced moment. The 4*d* DLs however, could initially promote CIDWM by an increase in the DW width that results from a decreased K_u^{eff} whilst θ_{SH} is initially modestly increased.

Since the CIDWM in the SAF case has no dependence on *D*, the enhancement in CIDWM results from an increased DW width, a decreased α and a modestly increased θ_{SH}. E.g. a very good correspondence between the SAF sample with a 1Å Pd DL and the reference SAF sample is found when the data for the reference sample is recalibrated using the increased Δ, the decreased α and the modestly increased H_{SHE} as shown in Figure S13b.

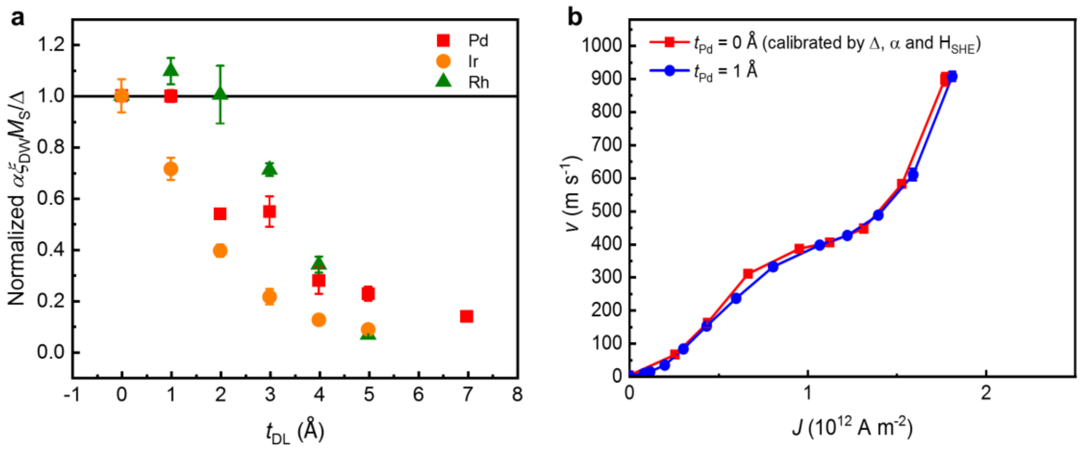

Figure S13. a) The DL thickness dependence of normalized $\frac{\alpha M_{\mathrm{S}}\xi_{\mathrm{DW}}}{\Delta}$ calculated from the DW mobility for the FM cases. The red squares, orange circles and green triangles represent Pd, Ir and Rh DL, respectively. b) *v*-*J* curve calibrated with DW width, α and θ_{SH} in the SAF case is compared with a Pd DL of 1 Å. Red squares and blue circles correspond to the calibrated reference SAF sample and the 1 Å thick DL sample, respectively.

Section 8: Magnetization and *M*-H_z curves of SAF films and summaries of coercive fields in FM films

In order to obtain the M_r/M_s ratio, the out-of-plane *M*-H_z curves of the SAF samples with DLs are measured using a superconducting quantum interference device (SQUID) at room temperature. The measured data are shown in Figure S14. M_r and M_s are defined as the magnetization measured at fields of 0 kOe and 15 kOe, respectively, after a linear background was subtracted. We also measured the *M*-H_z curves of the FM samples: the coercive fields (H_C) are summarized as below. H_C drops quickly and then slowly increases as the Pd and Rh DL thicknesses are increased. For the Ir DL case there is a much faster increase after an initial decrease of H_C as soon as the DL is introduced.

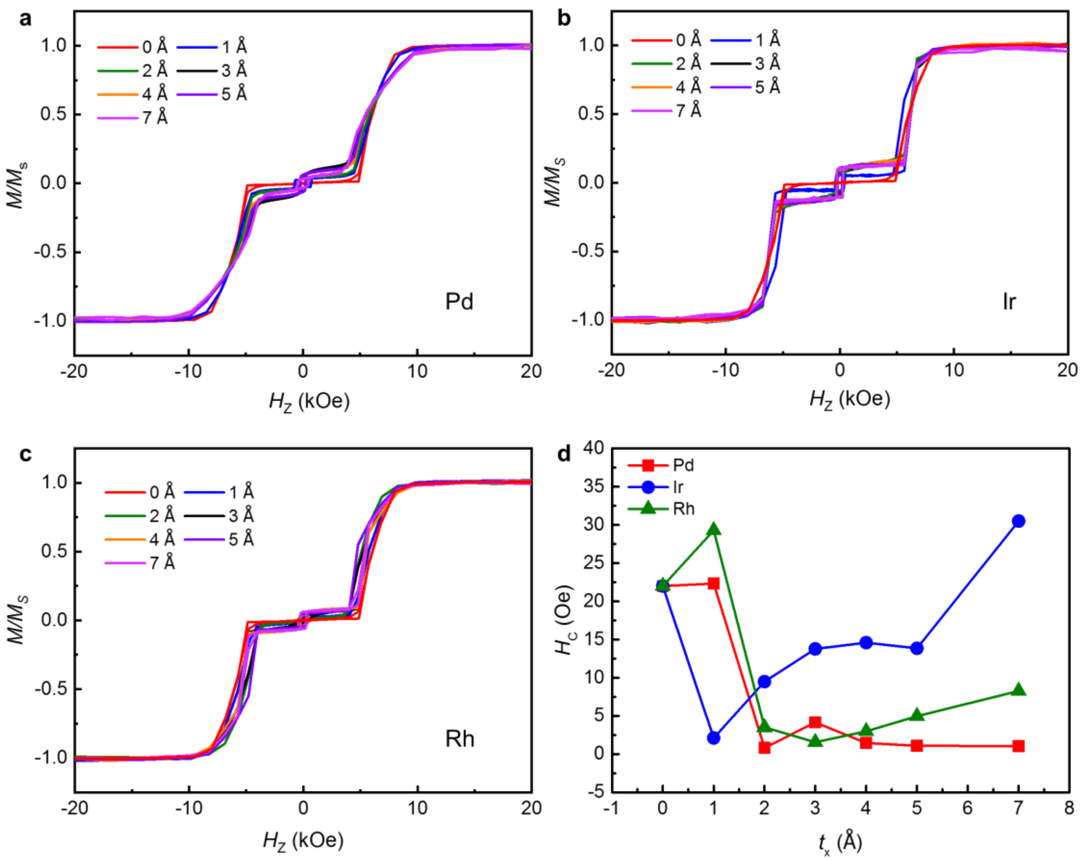

Figure S14 Out-of-plane *M*-H_z curves of SAF films with Pd (a), Ir (b), and Rh (c) DLs and summaries of coercive fields of SL films with DL thickness (d).

Section 9: Determination of the Gilbert damping parameter from optical Ferromagnetic Resonance (OFMR) measurements

The Gilbert damping parameter α of the FM sample with DLs is obtained by analyzing the OFMR spectra of the samples for different frequencies of the injected current (as shown in Figure S15a). An Agilent HP 83620B rf generator is used to provide the radio frequency current while a magnetic field is applied in-plane. α is then obtained from the linear fitting of the OFMR spectra linewidth against the frequency, as shown in Figure S15b. The values of α versus DL thickness are summarized in Figure S15c. Due to the limited magnitude of the applied in-plane field, samples with H_K^{eff} values that are too high could not be measured. In these cases, values of α from ref. [S2] are used (reference sample, and Ir DLs with thicknesses of 1, 2, 3, and 4 Å).

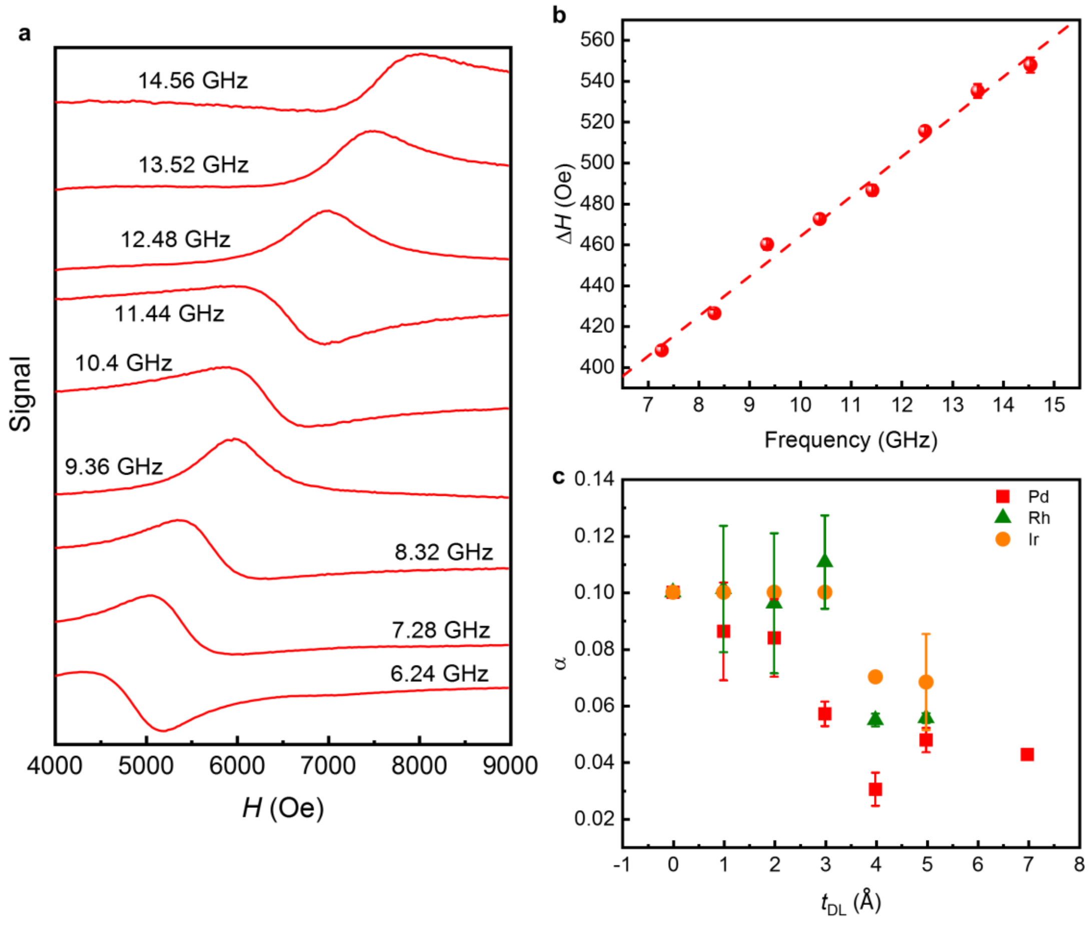

Figure S15 OFMR determined gilbert damping parameters. a) The OFMR spectra of the sample with a 4 Å Rh DL for various frequencies; b) The fitted linewidth of the OFMR spectra in Figure S15a plotted as a function of frequency; c) Summary of the damping parameters obtained for different DL materials and thicknesses.

Section 10: CIDWM in FM and SAF structures with Ru DLs

Ru DLs (not included in main text) were also investigated, as shown in Figure S16. The hexagonal structure of the Ru layer prevents coherent growth from the *fcc* Pt underlayer. The PMA becomes quickly weaker as the Ru DL thickness is increased. For the FM case the sample becomes multi-domain when the Ru DL exceeds 3 Å in thickness. Steady DW motion could be achieved solely for the 1 Å Ru DL case but with a very low efficiency.

In the SAF case, however, PMA could be well established owing to the very large anti-ferromagnetic coupling. The efficiency of the CIDWM decreases even faster than the Ir DL case when the Ru DL thickness is increased. When the Ru DL is thick enough (~ 5 Å), the DW moves opposite to the current injection direction with a very low efficiency. This is a typical behavior of STT driven DW motion. This behavior is similar to that observed for the Au DL case in earlier studies [Ref. S2] where the DMI and the proximity-induced magnetization (PIM) in the Pt layer are eliminated with the introduction of very thin Au DLs. The threshold current density is decreased with the introduction of the Ru DL in both the FM and SAF structures including the case with a dominant STT-DW driven mechanism. Such a scenario has the same origin, as discussed in the main text.

This control experiment has a very important implication with regard to the selection of the interfacial DL material: the *fcc* structure of the DL enables a coherent growth from the Pt underlayer, thus realizing stable chiral DWs, that is required to achieve CIDWM with high efficiency.

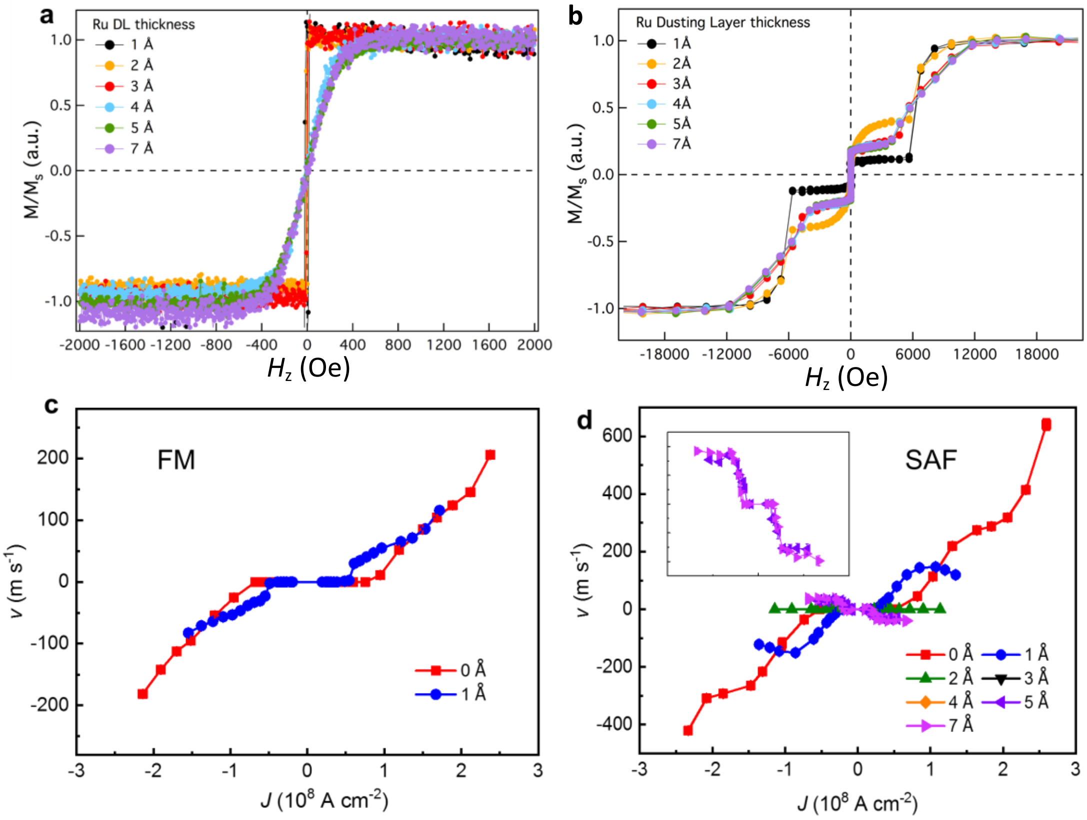

Figure S16 Out-of-plane *M*-H_z curves for FM (a) and SAF (b) structures with Ru DLs. CIDWM for the same FM (c) and SAF (d) structures. The inset to (d) is a magnified plot showing the STT-DW driven behavior for the 5 and 7 Å Ru DLs.

Section 11: Confirmation of interfacial effect of DL by a sandwiched HM layer

In order to confirm the interfacial nature of the DL effects, we have prepared a set of samples in which a dusting layer of fixed thickness (2 Å Pd) and composition is systematically moved spatially in the film normal direction from the interface into the interior of the Pt underlayer. These structures have the form: TaN(50 Å)/Ru(8.5 Å)/Co(1.5 Å)/Ni(7 Å)/Co(3 Å)/Pt(x Å)/Pd(2 Å)/Pt(15-x Å)/TaN(20 Å), where x is varied from 0~5 Å. As shown in Figure S17, almost as soon as the Pd DL has been moved away from the Co/Pt interface, large changes in uniaxial anisotropy energy and current induced domain wall motion occur, while the saturation magnetization only exhibits a small change. Indeed, when the Pd DL is moved just 3 Å away from the Co/Pt interface, the samples behave much like the reference sample without any DL (whose properties are indicated by the red dashed line in the Figure S17). These results clearly show that the DL is only effective when positioned at the Co/Pt interface, confirming the thesis in the main text.

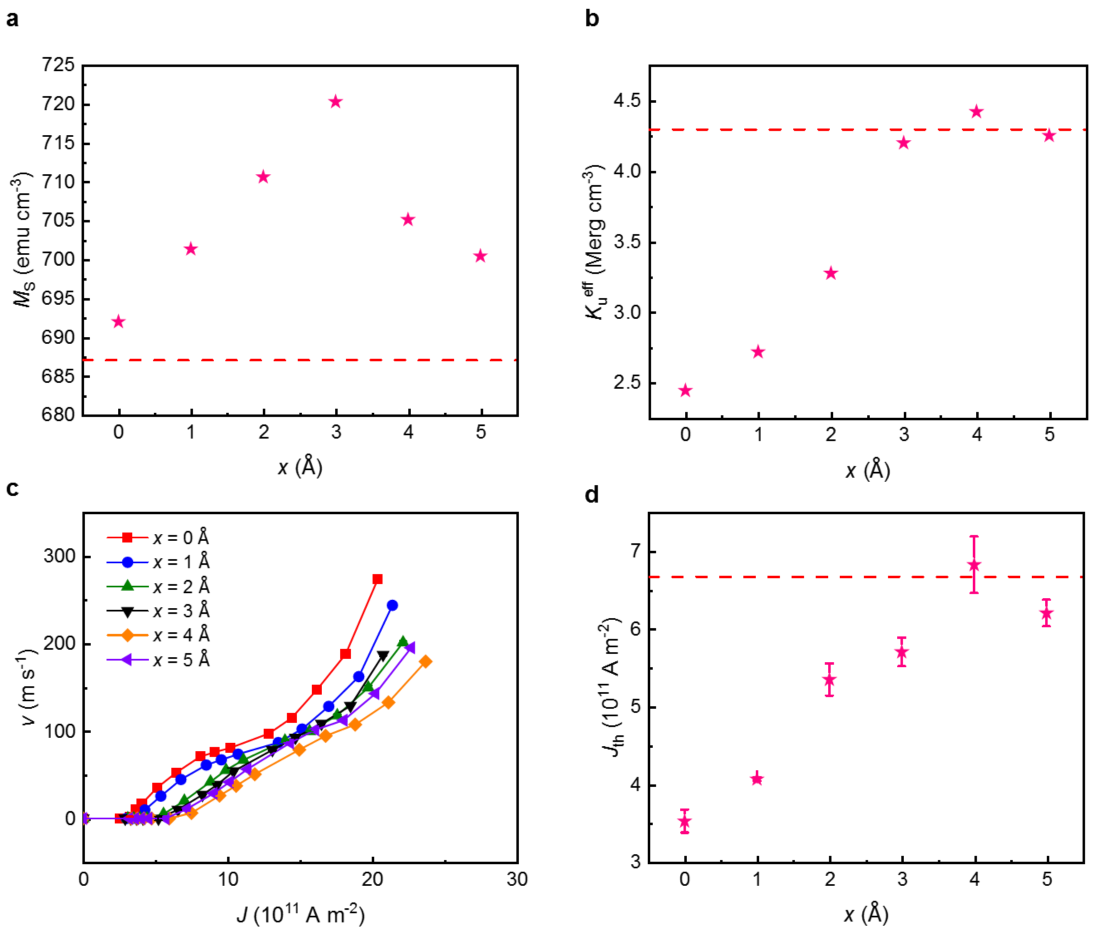

Figure S17: Influence of position of DL on magnetic properties and current induced domain wall motion in a FM structure. Saturation magnetization M_S, uniaxial anisotropy energy K_u^{eff}, current induced domain wall motion v, and threshold current density J_{th} for the set of structures: TaN(50 Å)/Ru(8.5 Å)/Co(1.5 Å)/Ni(7 Å)/Co(3 Å)/Pt(x Å)/Pd(2 Å)/Pt(15-x Å)/TaN(20 Å), for x varied from 0 to 5 Å. (a) M_S, (b) K_u^{eff}, and (d) J_{th} plotted versus x. The dashed lines indicate the

values of these three quantities measured on an identical reference FM structure without any DL. (c) *v* versus current density for x = 0 to 5 Å.

References

[S1] S. –H. Yang, K. –S. Ryu, S. S. P. Parkin. *Nat. Nanotechno*. **2015**, *10*, 221.

[S2] K. -S. Ryu, S. –H. Yang, L. Thomas, S. S. P. Parkin. *Nat. Commun*. **2014**, *5*, 3910.

[S3] A. Thiaville, S. Rohart, E. Jue, V. Cros, A. Fert. *Europhys. Lett*. **2012**, *100*, 57002.

[S4] A. P. Malozemoff, and J. C. Slonczewski. *Solid State Sci*. Academy press, New York, **1979**.

[S5] L. Caretta, M. Mann, F. Büttner, K. Ueda, B. Pfau, C. M. Günther, P. Hessing, A. Churikova, C. Klose, M. Schneider, D. Engel, C. Marcus, D. Bono, K. Bagschik, S. Eisebitt, G. S. D. Beach. *Nat. Nanotechno.* **2018**, 12, 1154

[S6] C. O. Avci, E. Rosenberg, L. Caretta, F. Büttner, M. Mann, C. Marcus, D. Bono, C. A. Ross, G. S. D. Beach. *Nat. Nanotechno.* **2019**, 14, 561
